# Adaptation mechanism of three *Impatiens* species to different habitats based on stem morphology, lignin and *MYB4* gene

**DOI:** 10.1186/s12870-024-05115-3

**Published:** 2024-05-24

**Authors:** Xin-Yi Li, Ze-Feng Li, Xiao-Li Zhang, Meng-Qing Yang, Pei-Qing Wu, Mei-Juan Huang, Hai-Quan Huang

**Affiliations:** Southwest Forestry University, College of Landscape Architecture and Horticulture Sciences, Southwest Research Center for Engineering Technology of Landscape Architecture (State Forestry and Grassland Administration), Yunnan Engineering Research Center for Functional Flower Resources and Industrialization, Research and Development Center of Landscape Plants and Horticulture Flowers, Kunming, Yunnan, 650224 China

**Keywords:** *Impatiens*, Stem morphological anatomy, *MYB4* gene, Expression analysis, Lignin

## Abstract

**Background:**

*Impatiens* is an important genus with rich species of garden plants, and its distribution is extremely extensive, which is reflected in its diverse ecological environment. However, the specific mechanisms of *Impatiens*’ adaptation to various environments and the mechanism related to lignin remain unclear.

**Results:**

Three representative *Impatiens* species,*Impatiens chlorosepala* (wet, low degree of lignification), *Impatiens uliginosa* (aquatic, moderate degree of lignification) and *Impatiens rubrostriata* (terrestrial, high degree of lignification), were selected and analyzed for their anatomical structures, lignin content and composition, and lignin-related gene expression. There are significant differences in anatomical parameters among the stems of three *Impatiens* species, and the anatomical structure is consistent with the determination results of lignin content. Furthermore, the thickness of the xylem and cell walls, as well as the ratio of cell wall thickness to stem diameter have a strong correlation with lignin content. The anatomical structure and degree of lignification in *Impatiens* can be attributed to the plant's growth environment, morphology, and growth rate. Our analysis of lignin-related genes revealed a negative correlation between the *MYB4* gene and lignin content. The *MYB4* gene may control the lignin synthesis in *Impatiens* by controlling the structural genes involved in the lignin synthesis pathway, such as *HCT*, *C3H*, and *COMT*. Nonetheless, the regulation pathway differs between species of *Impatiens*.

**Conclusions:**

This study demonstrated consistency between the stem anatomy of *Impatiens* and the results obtained from lignin content and composition analyses. It is speculated that *MYB4* negatively regulates the lignin synthesis in the stems of three *Impatiens* species by regulating the expression of structural genes, and its regulation mechanism appears to vary across different *Impatiens* species. This study analyses the variations among different *Impatiens* plants in diverse habitats, and can guide further molecular investigations of lignin biosynthesis in *Impatiens*.

**Supplementary Information:**

The online version contains supplementary material available at 10.1186/s12870-024-05115-3.

## Introduction

The emergence of lignin is one of the most important alterations in the evolution of aquatic plants into terrestrial environments, enabling them to adapt to the terrestrial ecological conditions. Lignin plays a crucial role in enhancing plants' mechanical strength, water transport and resistance to external bacteria invasion. In addition, it improves plants' defense capacity against biological and abiotic stresses [1–4]. Lignin comprises a significant proportion of plant cell structure, contributing about a third of woody plant biomass on land [5]. Lignin primarily consists of three monomers—*p*-hydroxyphenyl (H-lignin), guaiacyl (G-lignin) and syringyl lignin (S-lignin)—that are polymerized. Some atypical monomers such as catechyl and 5-hydroxyguaiacyl (5H) have also been detected [6, 7].

The content and composition of the three lignin monomers are different in different plants. In general, G and S-lignin are dominate in dicots, H-lignin is less pronounced. In monocots plants, H-lignin is higher than in dicots. Gymnosperms have higher levels of both G-lignin and H-lignin [8, 9]. The process of lignin biosynthesis involves 11 key enzymes, including phenylalanine ammonia-lyase (PAL), cinnamate 4-hydroxylase (C4H), 4-coumarate-CoA ligase (4CL) from the non-specific lignin biosynthesis pathway, as well as shikimate/quinate hydroxycinnamoyl transferase (HCT), coumarate 3-hydroxylase (C3H), caffeoyl shikimate esterase (CSE), ferulate 5-hydroxylase (F5H), caffeic acid 3-O-methyltransferase (COMT), caffeoyl-CoA O-methyltransferase (CCoAOMT), cinnamoyl-CoA reductase (CCR) and cinnamyl alcohol dehydrogenase (CAD) are specific enzymes involved in the biosynthesis pathway of lignin [10, 11].

The regulation of plant lignin synthesis is governed through a three-level network. NAC transcription factors serve as the first-order switches, *AtMYB46* and *AtMYB83* are the second-order switches [12–14]. Additional MYB transcription factors, such as *AtMYB20*, *AtMYB42*, *AtMYB43* and *AtMYB85* are the third-order regulatory genes [15, 16]. These transcription factors have the capability to activate the expression of lignin synthesis genes which, in turn, further influences the formation of plant secondary cell walls. It has been reported that MYB transcription factors inhibiting lignin synthesis can be found in most plants, such as *MYB4* in *Arabidopsis thaliana* [17, 18], *CmMYB8* in *Chrysanthemum morifolium* [19], and *EgMYB1* in *Eucalyptus gunnii* [20], which can negatively regulate lignin synthesis.

The MYB4 transcription factor possesses R2 and R3 domains specific to the MYB family and belongs to the R2R3-MYB. Overexpression of *AtMYB4* in *Nicotiana tabacum* resulted in decreased in tobacco growth and lignin content [17, 21]. Similarly, overexpression of *PvMYB4* in *Panicum virgatum* led to a decrease in lignin content [22]. Transgenic *Arabidopsis thaliana* plants that overexpress *NnMYB4* exhibited a significant reduction in stem lignin content, suggesting that *NnMYB4* from *Nelumbo nucifera* may function as a transcriptional inhibitor of lignin synthesis [23]. Meanwhile, overexpressing *BpMYB4* in *Arabidopsis thaliana* promoted stem growth while reducing lignin deposition, indicating that *BpMYB4* inhibits lignin biosynthesis [24]. It can be seen that the transcription factor MYB4 plays a negative role in regulating lignin in most plants.

The family Balsaminaceae contains of only two genera: single species of *Hydrocera* and *Impatiens*. *Impatiens* is mainly distributed in southwest China, especially in Yunnan. The *Impatiens* species are mostly herbaceous plants with rich colors, peculiar flower shapes, and fleshy stems. Most are annuals, though a few are perennials. Additionally, there are also shrub-like species, characterized by hard and lignified stems. Currently, the research of *Impatiens* species focuses on cultivation management, tissue culture, flower organ development, plant disease, molecular biology and evolution [25–29]. For instance, Li [26] explored the related development mechanism of the spur of *I. uliginosa* with important ecological value. Janssens [30] reconstructed the phylogenetic tree of 85 *Impatiens* species using the chloroplast gene *atpB-rbcL* and the nuclear gene *ITS* and validated the sisterhood relationship between *Impatiens* and *Hydrangea*.

*Impatiens* species inhabit tropical and subtropical regions and typically thrive in high-humidity habitats, including forests and streams, while some species are adapted to grow in arid areas [31]. Due to its complex and diverse habitats, the genus *Impatiens* has a great diversity, including rich ecotypes, such as herbs, subshrubs, woody plants [32] and erect or creeping flora, as well as rich growing environments, such as aquatic, moist and terrestrial. Lens [32] discovered significant variation in the stems of *Impatiens* species and explored the evolutionary relationship between herbaceous and woody forms in *Impatiens* by studying their anatomical structure. Stem plays an important role in the growth and development of plants. Stem xylem is related to plant growth rate and drought adaptation [33–35]. Słupianek [36] found that the differences in the vessel diameter in the secondary xylem of *Salix polaris* stems is mainly related to the humidity of the habitat. These researches highlight the significant role that stems play in plant adaptation to their environment. The diversity among stems of *Impatiens* species provides excellent resources for studying the origin and evolutionary adaptation of *Impatiens* to different environments. *Impatiens uliginosa* thrives in aquatic regions, with upright and hollow stems. *Impatiens chlorosepala* is born in the moist area, it has small and solid fleshy stems that creep as they grow. *Impatiens rubrostriata* grows in sparse forests or shrub grasslands with solid stems and relatively high lignification.

In this study, *I. uliginosa*, *I. rubrostriata* and *I. chlorosepala* were used as experimental materials. We aimed to analyze the differences in stem tissue among these three *Impatiens* species with varying lignification degrees in different habitats. This was achieved through morphological anatomy analysis, as well as the determination of stem lignin content and composition, MYB4 gene cloning, gene sequence structure, systematic evolution, and qRT-PCR analysis. This study can not only contributes to understanding the adaptation mechanism of *Impatiens* species to different habitats but also provide essential data and theoretical support for investigating the evolutionary mechanism of *Impatiens* species from aquatic to terrestrial environments, because lignin has an important influence on the evolutionary process of plants from aquatic to terrestrial [37, 38]. Furthermore, it helps to provide basic data and theoretical support for the study of the adaptation mechanism of *Impatiens* to aquatic and terrestrial habitats, and is conducive to further improving the characteristics and resistance of *Impatiens* through the use of genetic engineering and other technologies, to promote the development and utilization of *Impatiens* and enrich the garden landscape.

## Materials and methods

### Experimental materials

**Fig. 1:**
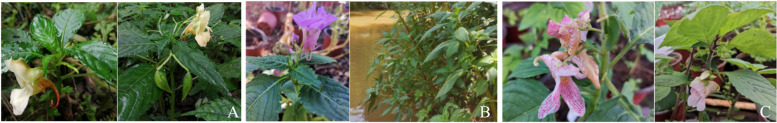
Test materials. **A**:* I*. *chlorosepala*. **B**: *I*. *uliginosa.***C**: *I*. *rubrostriata*

Three *Impatiens* species were collected from the field and planted in the greenhouse of Southwest Forestry University arboretum to simulate the growing environment for routine water and fertilizer management. *I*. *chlorosepala* grows in wet forests and streams and has creeping stems, *I*. *uliginosa* grows well in water and has upright stems, *I*. *rubrostriata* thrives in dry hillside rock and also has upright stems (Fig. 1). The anatomy of stem morphology selected the stem base of 5–10 cm above the ground that grows strong after flowering and maturity for the experiment. Lignin content was determined using whole stems of three *Impatiens* species. The cloning of the *MYB4* gene was utilized to select healthy plant stems, which grow until flowering and maturity. The healthy root tip (5 cm), the stem base 5 cm above the ground, and the fourth mature leaf from top to bottom were selected for qRT-PCR.

### Histochemical analysis

The paraffin sectioning method was modified based on Tong et al. [39]. The collected samples were fixed in FAA solution and then dehydrated by alternating 70%, 85%, 95%, 100% ethanol, and 1/2 absolute ethanol + 1/2 pure xylene (v/v). After washing with xylene, the stems were embedded in paraffin. Paraffin sections of 10 μm were cut with a slicer, dried and stained with saffron fixation, sealed with gum, observed and photographed with the Eclipse Ci-L microscope. The anatomical structures of three *Impatiens* species stems were determined using Caseviewer software. The corresponding structural indices of the main determination are listed in Table S1.

### Determination of total lignin content

The stems of three *Impatiens* species were dried in an oven at 37 ℃, weighed with an analytical balance until the stems dried to a constant weight, ground into fine powder with a mortar, sieved with a 40-mesh sieve and stored in a dryer for later use. The lignin content consisted of acid-soluble and acid-insoluble lignin, and the specific determination method referred to the method of Wu et al. [40].

0.5000 g (W1) of dried stem tissue that has passed through a 40-mesh sieve was weighed. The sample was extracted with benzene-ethanol (67:33, v/v) in a Soxhlet extractor for 4 h. After air drying, the powder was transferred into a 250.0 mL triangular flask and treated with 72% concentrated sulfuric acid at 30℃ and 120 r/min for 1.5 h. The hydrolysate was filtered using a G3 crucible filter. The residue was washed with distilled water until it reached a neutral pH. The filtrate was then adjusted to a volume of 250.0 mL and the concentration of sulfuric acid was 2.88%. The residue and filter were dried at 80℃ until a constant weight was achieved (W2). The filter and residue were placed on a scale and transferred to a muffle furnace. The mixture was heated at 200℃ for 30 min and then ashed at 575 ± 25℃ for 4 h. The mixture should be removed and allowed to cool in a dryer for 30 min. Weigh the sample to obtain W3 (ash + filter). 1 mL of the 250.0 mL filtrate was taken and diluted ten times with 2.88% sulfuric acid (depending on the sample). The absorbance was measured at 205 nm using an ultraviolet spectrophotometer, ensuring that it fell between 0.2 and 0.7. The dilution multiple was recorded as D. The control used was 2.88% sulfuric acid. Repeat each sample in triplicate.

The content of acid-insoluble lignin is: AIL% = (W2-W3) × 100/W1. The content of acid-soluble lignin is: ASL% = A × D × V/(1000 × K × W1) × 100. The content of total lignin in the sample is: Lignin(%) = AIL(%) + ASL(%). (A: absorption value; D: dilution multiple; V: total volume of filtrate; K: absorption coefficient of acid-soluble lignin (110); W1: sample quality.)

### Lignin monomer content and composition analysis

The material treatment method was identical to that used to determine the lignin content. Alkaline nitrobenzene oxidation method was used to determine the lignin monomer content in the stems of three *Impatiens* species by high-performance liquid chromatography. The specific steps were taken from Xu et al. [41].

0.1000 g of stem tissue powder was weighed (passed through a 40 mesh sieve), extracted for 6 h in a Soxhlet extractor containing benzene/ethanol (67:33, V/V) solution and air dried to constant weight to obtain cell wall residue (CWR). Weigh 50.0 mg of CWR, place in a sealed PTFE tank (25.0 mL) and add 5.0 mL of 2.0 mol/L NaOH solution, 0.5 mL of nitrobenzene and a rotor. After tightening and sealing the whole apparatus, it was placed in a constant temperature oil bath at 170℃ for 3.5 h at a rotation speed of 15 r/ min. After the reaction, the sealed vessel was rapidly cooled with cold water. The reaction mixture in the sealed vessel was transferred to a 100.0 mL triangular flask. Add a certain amount of internal standard to the triangular flask, the internal standard is ethyl vanillin and the concentration is 4.0 mg/mL (2.0 mol/L NaOH configuration). The reaction mixture was extracted three times with 30.0 mL of dichloromethane/ethyl acetate mixture (1:1, V/V). Retain the aqueous phase (in this step the upper layer of the extract is retained and the lower layer contains nitrobenzene derivatives). Adjust the pH of the aqueous phase to 3–4 with 6.0 mol/L HCl and shake well. Extract again three times with 30.0 mL dichloromethane/ethyl acetate mixture (1:1, V/V). This step retains the bottom layer of the extract. The organic phase was collected and evaporated at 40℃ to obtain a solid residue. The residue was redissolved with 5.0 mL of chromatographically pure methanol, filtered through a 0.22 µm membrane filter, and 20.0µL was taken for HPLC detection.

### Gene cloning

Screen *MYB4* genes from three *Impatiens* species transcriptome data and carry out subsequent experiments. Total RNAs were extracted from mature stems of three *Impatiens* species using the Plant RNA Kit R6827 of Omega Bio-Tek, and then the first strand of cDNAs were synthesized using the TransScript® One-Step gDNA Removal and cDNA Synthesis SuperMix of TransGen Biotech. Using first strand of cDNAs as templates, three *Impatiens* species’ full-length *MYB4* genes were amplified. The primers used are indicated in Table S2. The PCR reaction system consisted of 10 × EasyTaq Buffer(Mg^2+^) 2.4 μL, dNTP 1.6 μL, EasyTaq DNA Polymerase 0.2 μL, upstream and downstream primers 1 μL each, cDNA template 1 μL, ddH_2_O 12.8 μL, total volume of 20 μL. The PCR reaction procedure is: 95℃ 5 min; 95℃ 50 s, 56℃ 30 s, 72℃ 1 min, 35 cycles; 72℃ 10 min; 4℃ 10 min.

According to the DNA extraction kit DP3111 of Bioteke, the DNAs of mature stems of three *Impatiens* species were extracted and the genomic DNAs of *MYB4* genes of three *Impatiens* species were cloned using them as templates. The PCR reaction system and procedure were identical to those described above.

### Fluorescence quantitative analysis

Total RNAs were extracted from the roots, stems, and leaves of three *Impatiens* species. *Actin* gene was used as the reference gene. Primers for qRT-PCR analysis were designed according to the obtained *MYB4* sequences of three *Impatiens* species. Primers of structural genes *COMT*, *F5H*, *C3H* and *HCT* in three *Impatiens* species were designed in highly conserved fragments, as show in Table S3.

Using three-step qRT-PCR, the expression levels of different copies of the *MYB4* gene in roots, stems and leaves of three *Impatiens* species were detected to analyze. According to the expression levels of different copies of *MYB4* gene in different parts of *Impatiens*, the copies with strong function in the stems of three *Impatiens* species were screened, and the expression levels of *MYB4* gene in three *Impatiens* species were analyzed using universal reference primers and the expression levels of structural gene in the stems of three *Impatiens* species were also analyzed. The reaction system of qRT-PCR is: qPCR SYBR Green Master Mix (Yisheng Company) 10 μL, ddH_2_O 8.2 μL, F primer 0.4 μL, R primer 0.4 μL, cDNA 1 μL, total volume 20 μL. Amplification procedure: 95℃ 5 min; 95℃ 15 s, 60℃ 30 s, 72℃ 1 min, 40cycles; 95℃ 15 s; 60℃ 1 min; 95℃ 15 s. For cloning and the relative expression levels data of the *MYB4* genes from *I. chlorosepala*, see Li et al. [42].

## Results

### Structural composition of stems of three *Impatiens* species

**Fig. 2:**
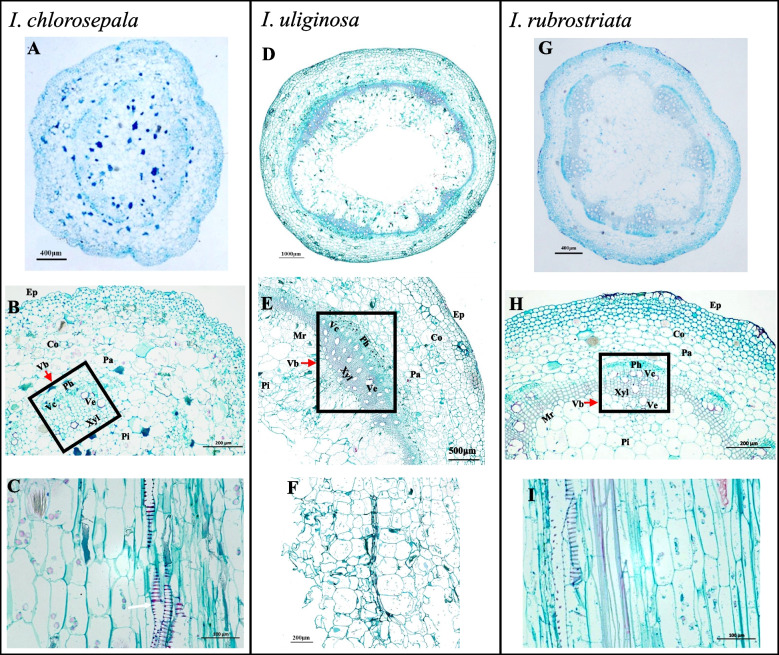
Transverse (**A**, **B**, **D**, **E**, **G**, **H**) and longitudinal (**C**, **F**, **I**) light microscope stem sections of *Impatiens* species. **A** and **B** Transverse section of *I. chlorosepala*. **C** Tangential section of *I. chlorosepala*. **D** and **E** Transverse section of *I. uliginosa*. **F** Tangential section of *I. uliginosa*. **G** and **H** Transverse section of *I. rubrostriata*. **I** Tangential section of *I. rubrostriata*. Ep: epidermal cells, Co: cortex, Pa: parenchyma cells, Vb: vascular bundle, Ve: vessels, Vc: cambium, Mr: medullary ray, Xyl: xylem, Pi: pith, Ph: phloem

The anatomical structures of the stems of three *Impatiens* species are basically the same, consisting of epidermis, cortex, phloem, cambium, xylem, parenchyma, and pith from outside to inside (Fig. 2 A, D, G). The epidermis of three *Impatiens* species is composed of one layer of cells. While *I*. *uliginosa* and *I*. *rubrostriata* have regularly arranged and neatly organized epidermal cells, *I*. *chlorosepala* has twisted and irregular epidermal cells (Figure S1). The cortex is composed of parenchyma cells and has multiple layers of cells. The cortex lies adjacent to the xylem and phloem, and the xylem of *I*. *chlorosepala* is smaller, and the vascular bundle area is not obvious. The vascular bundles of* I*. *uliginosa and I*. *rubrostriata* are clearly visible, with obvious xylem, and relatively larger vessel diameter. The parenchyma cells within the pith of three *Impatiens* species are of large size. The arrangement of parenchyma cells in the pith of *I*. *chlorosepala* is disordered, while fewer parenchyma cells exist in the pith of *I. uliginosa* and the middle part of its pith is hollow, *I. rubrostriata* boasts a close and orderly arrangement of parenchyma cells (Fig. 2 B, E, H; Figure S2).

The cells of *I. chlorosepala* are irregular, arranged in disorder and loose. However, the cells of *I. uliginosa* are mostly regular square and closely arranged. *I. rubrostriata* cells are mostly elongated, which are closely arranged layer by layer from epidermis to xylem, and are more enlarged in the pith parenchyma (Fig. 2 A, B, D, E, G, H). The stem composition of three *Impatiens* species is nearly identical, while the cell shapes differ significantly among them. Additionally, all three *Impatiens* species have scalariform vessels (Fig. 2 C, F, I).

### Comparison of anatomical parameters of stems of three *Impatiens* species

The epidermis of *I*. *uliginosa* is the thickest, which is significantly different from *I*. *chlorosepala* and *I*. *rubrostriata*. Additionally, *I. uliginosa* has the largest stem diameter among the three species at the same growth stage. The thickness of cortical cells, epidermis, vessel diameter, pith diameter, xylem thickness, phloem thickness, and cell wall are also significantly different from *I*. *chlorosepala* and* I*. *rubrostriata*. All other indicators do not show any noticeable differences between the species. The above results may be attributed to the stem diameter of *Impatiens* species.

However, among three *Impatiens* species, the ratio of cell wall thickness to stem diameter (Cw/D), ratio of xylem thickness to stem diameter (Xyl/D), ratio of vascular bundle area to stem cross-sectional area (Vb/S) and ratio of xylem area to stem cross-sectional area (Sxyl/S) are the highest in *I*. *rubrostriata* compared to *I*. *uliginosa* and *I*. *chlorosepala*, which is a significantly distinction among the three *Impatiens* species. The results show that the lignification degree of the erect semi-woody *I*. *rubrostriata* is indeed higher than that of the erect *I*. *uliginosa* and the creeping *I*. *chlorosepala*, indicating a strong correlation between the lignification degree of *Impatiens* species and their stems thickness, cell wall thickness, as well as the ratios of vascular bundle area to stem cross-sectional area and xylem area to stem cross-sectional area (Table 1).

**Table 1:** Anatomical parameters of the stems of three *Impatiens* species

**Index**	Species	*I*. *chlorosepala*	*I*. *uliginosa*	*I*. *rubrostriata*
epidermal cell layer		1	1	1
diameter of stem (µm)		2587.680 ± 108.400b	9036.600 ± 389.100a	2856.140 ± 74.676b
cortical cell thickness (µm)		507.330 ± 93.820b	1143.840 ± 201.520a	346.060 ± 40.660b
epidermal thickness (µm)		14.280 ± 4.300b	28.360 ± 3.570a	18.540 ± 4.420b
vascular bundle number		6	14	10
vascular bundle area (mm^2^)		0.430 ± 0.030b	3.42 ± 0.040a	1.160±0.020b
diameter of vessel (µm)		41.620 ± 3.120b	68.720 ± 12.730a	52.850 ± 4.120b
diameter of pith (µm)		1165.380 ± 31.810b	5911.320 ± 746.650a	1720.510 ± 219.890b
xylem thickness (µm)		79.400 ± 9.430c	356.280 ± 35.450a	237.420 ± 124.500b
cambium thickness (µm)		49.830 ± 13.220a	59.820 ± 25.070a	52.020 ± 14.370a
phloem thickness (µm)		31.700 ± 10.440b	108.060 ± 26.600a	38.630 ± 13.010b
cell wall thickness (µm)		0.850 ± 0.071c	3.800 ± 1.366a	1.620 ± 0.240b
xylem area (mm^2^)		0.030 ± 0.004c	0.420 ± 0.022a	0.070 ± 0.004b
stem cross-sectional area (mm^2^)		0.530 ± 0.045b	6.410 ± 0.546a	0.640 ± 0.034b
ratio of xylem thickness to stem diameter-Xyl/D (%)		3.07% ± 0.006b	3.94% ± 0.005b	8.31% ± 0.040a
ratio of cell wall thickness to stem diameter-Cw/D (%)		0.03% ± 0.003b	0.04% ± 0.020b	0.06% ± 0.008a
ratio of vascular bundle area to stem cross-sectional area-Vb/S (%)		4.30% ± 0.750b	5.36% ± 0.660b	18.10% ± 1.370a
ratio of xylem area to stem cross-sectional area-Sxyl/S (%)		5.48% ± 0.700b	6.58% ± 0.500b	11.40% ± 0.700a

Different lowercase letters indicate significant differences (*P* < 0.05)

### Correlation of stem morphology and anatomical structure of three *Impatiens* species

The results of the correlation analysis indicate that the stem diameter of *I*. *chlorosepala* has a positive correlation with both cell wall thickness and xylem area, as well as a negative correlation with all other indexes. Additionally, xylem thickness is positively correlated with Xyl/D and Sxyl/S, the cell wall thickness is positively correlated with Cw/D, and the vascular bundle area is positively correlated with Vb/S and Sxyl/S (Table S4). In *I*. *uliginosa*, stem diameter exhibits a positive correlation with xylem area and vascular bundle area, and a negative correlation with Cw/D, Xyl/D, Vb/S and Sxyl/S. Xylem thickness demonstrates a significant positive correlation with Xyl/D, while cell wall thickness shows a positive correlation with Cw/D and Sxyl/S (Table S5). In *I*. *rubrostriata*, the stem diameter is negatively correlated with Vb/S and Sxyl/S, the cell wall thickness is positively correlated with Cw/D, the xylem thickness is positively correlated with Cw/D and Sxyl/S, and Sxyl/S is positively correlated with Cw, Sxyl, Vb, Cw/D, Xyl/D and Vb/S (Table S6). We speculate that among the three *Impatiens* species, the stem diameter may have a certain influence on the anatomical structure of the stem, but Sxyl/S is mainly related to the xylem thickness, vascular bundle area and cell wall thickness.

### Difference analysis of total lignin content of three *Impatiens* species

The lignin content of three *Impatiens* species stems is significantly different. The acid-soluble lignin content of *I*. *chlorosepala* is 3.6%, that of *I*. *uliginosa* and *I*. *rubrostriata* is 9.78% and 9.46% respectively, which is significantly different from that of *I*. *chlorosepala*. The acid-insoluble lignin content in three *Impatiens* species is comparatively low, among which *I*. *chlorosepala* and *I*. *uliginosa* have similar values at 0.61% and 0.65%, respectively, while *I*. *rubrostriata* has a significantly higher content of 1.27% (Table 2). The highest content of total lignin was *I*. *rubrostriata* (10.73%), followed by *I*. *uliginosa* (10.43%) and *I*. *chlorosepala* (4.21%). Among the three *Impatiens* species, the erect semi-woody *I*. *rubrostriata* has the highest lignification degree, while the creeping herb *I*. *chlorosepala* has the lowest lignification degree. These results are consistent with the findings of stem anatomy analysis.

**Table 2:** Lignin content of the stems of three *Impatiens* species

**Lignin content**	Species	*I*. *chlorosepala*	*I*. *uliginosa*	*I*. *rubrostriata*
acid-soluble lignin (%)		3.60% ± 0.065c	9.78% ± 0.078a	9.46% ± 0.144b
acid-insoluble lignin (%))		0.61% ± 0.017b	0.65% ± 0.019b	1.27% ± 0.099a
Total lignin (%)		4.21% ± 0.015c	10.43% ± 0.009b	10.73% ± 0.189a

Different lowercase letters indicate significant differences (*P* < 0.05)

### Analysis on the difference of lignin monomer content in the stem of three *Impatiens* species

The determination of lignin monomer content of three *Impatiens* species shows that *I*. *chlorosepala* has the lowest levels of H and G-lignin and lacks S-lignin, which may be related to its creeping growth habit. There is no significant difference in S-lignin between *I*. *uliginosa* and *I*. *rubrostriata*, but H-lignin and G-lignin of *I*. *rubrostriata* are significantly higher than *I*. *uliginosa*. These findings further support the conclusion that *I*. *rubrostriata* has the highest degree of lignification in its stems (Table 3; Figure S3).

**Table 3:** Lignin monomer content of the stems of three *Impatiens* species

**Content**	Species	*I*. *chlorosepala*	*I*. *uliginosa*	*I*. *rubrostriata*
H (mg/mL)		0.00776 ± 0.0001b	0.00804 ± 0.0002b	0.01628 ± 0.0010a
G (mg/mL)		0.00248 ± 0.0009c	0.04685 ± 0.0014b	0.06243 ± 0.0012a
S (mg/mL)		0	0.00342 ± 0.0004a	0.00346 ± 0.0004a

Different lowercase letters indicate significant differences (*P* < 0.05)

### Correlation analysis between total lignin content and morphological anatomy of three *Impatiens* species

Through the correlation analysis between the lignin content and the anatomical morphological indexes of three *Impatiens* species stems, it was found that the lignin content was positively correlated with the anatomical structure of stems, and the correlation coefficient with xylem thickness, cell wall thickness and Cw/D was higher (Table S7). This implies that the higher the Sxyl/S in morphological anatomy, the higher the lignin content and the lignification degree of *Impatiens* species’ stems.

### Cloning and sequence analysis of *MYB4 *gene from three *Impatiens* species

Three copies of *MYB4* genes are present in *I. chlorosepala* and *I. uliginosa*, whereas only two copies are found in *I. rubrostriata*. The target band for the gene is 600–900 bp, and they were named as *IcMYB4-1*, *IcMYB4-2*, and *IcMYB4-3* for *I. chlorosepala*, *IuMYB4-1*, *IuMYB4-2*, and *IuMYB4-3* for *I*. *uliginosa*, *IrMYB4-1* and *IrMYB4-2* for *I*. *rubrostriata*. The genomic DNA bands of *MYB4* genes in three *Impatiens* species range from 700–1000 bp (Table S8; Figure S4, S5, S6).

Using the Expasy website (http://expasy.org/tools/), the amino acid sequences encoded by *MYB4* genes in three *Impatiens* species were predicted. The results indicate that all MYB4 proteins are hydrophilic and unstable (Table S9), and all MYB4 proteins are mainly located in the nucleus (Table S10). The MYB4 proteins of three *Impatiens* species all contain the SANT domain and the DNA binding site of MYB, indicating that they belong to the MYB family. Moreover, the MYB4 proteins also contain a stable PLN03091 domain, which is basically consistent with the conserved domain of MYB4 proteins in other plants. Hence, it is speculated that the *MYB4* genes of three *Impatiens* species belong to the R2R3-MYB subfamily (Figure S7, S8, S9).

Multi-sequence comparison analysis of MYB4 proteins of three *Impatiens* species and other species revealed high conservation of all genes within the MYB family-specific domain. There are conserved R2 and R3 DNA binding domains at the N-terminal, but there are considerable variations at the C-terminal. Additionally, the inhibitory conserved motif EAR is present (Figure S10).

**Fig. 3:**
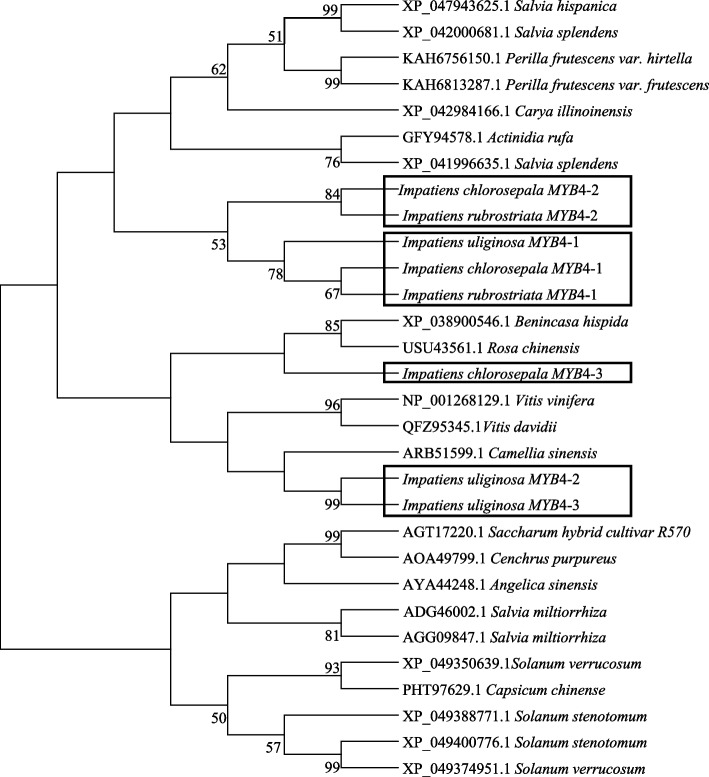
Phylogenetic tree of MYB4 of three *Impatiens* species (NJ)

The phylogenetic tree was constructed by NJ method with the MYB4 protein of three *Impatiens* species and its homologous MYB4 amino acid sequence, using MEGA7.0 software. The results show that the phylogenetic tree is divided into three branches. Three *MYB4-1* genes of *I. chlorosepala*, *I. uliginosa*, and *I. rubrostriata* cluster on a small branch, indicating a closer genetic relationship. *I. rubrostriata* and *I. chlorosepala*'s MYB4-2 get together, which is supposed to be a orthologous relationship. These genes are grouped into a big branch with plants including *Actinidia rufa*, *Salvia splendens*, *Salvia hispanica*, *Vitis vinifera*, *Carya illinoinensis*, and *Perilla frutescens*. *I. uliginosa*'s IuMYB4-2 and IuMYB4-3 are grouped together in another branch, which is also supposed to be a orthologous relationship. However, IcMYB4-3 from *I. chlorosepala* is clustered into a small branch alone, and it was conjoined with *Rosa chinensis* and *Benincasa hispida*, indicating orthologous. The above genes and plants such as *Camellia sinensis* and *Vitis davidii* are grouped into the second branch. The third branch comprises *Angelica sinensis*, *Salvia miltiorrhiza*, *Solanum stenotomum* and *Capsicum chinense*, all of which are annual or perennial herbs (Fig. 3). According to the phylogenetic tree, it can be inferred that the *MYB4* genes in three *Impatiens* species are closely related to those in some herbs and woody plants. Furthermore, they may have similar functions with the genes in other plants that are grouped together.

### Expression analysis of *MYB4 *gene in three *Impatiens* species

**Fig. 4:**
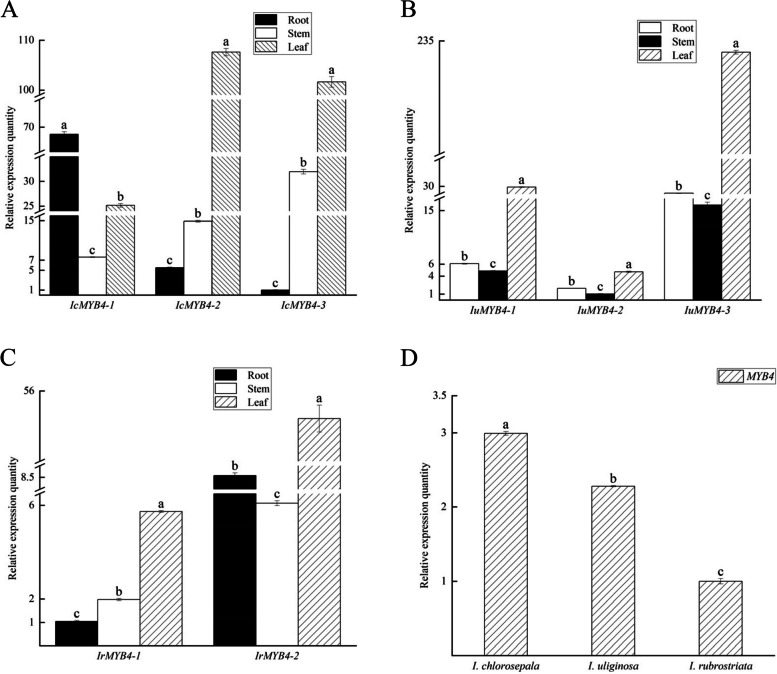
The expression pattern of *MYB*4 in different parts of three *Impatiens* species. **A** The expression pattern of *MYB*4 in different parts of *I. chlorosepala*. **B** The expression pattern of *MYB*4 in different parts of *I. uliginosa*. **C** The expression pattern of *MYB*4 in different parts of *I. rubrostriata*. **D** Relative expression of *MYB*4 gene in the stems of three *Impatiens* species. Different lowercase letters indicate significant differences (*P* < 0.05)

*MYB4* was expressed in the stems of three *Impatiens* species. The expression level was highest in *I. chlorosepala*, which had the lowest lignin content, followed by *I. uliginosa*, and the expression level in *I. rubrostriata* with the highest lignin content was the lowest (Fig. 4D). Combined with the determination and anatomical data on the lignin content in the stems of three *Impatiens* species, it is suggested that *MYB4* gene may has a negative regulatory impact on the synthesis of lignin in *Impatiens*.

Three *IcMYB4* genes were expressed in the roots, stems, and leaves of *I*. *chlorosepala* (Fig. 4A). *IcMYB4-1* had the lowest expression level in the stem of *I*. *chlorosepala*, followed by the leaf, and the highest expression level in the root. *IcMYB4-2* had the highest expression in leaves, about eight times that in stems, and the lowest expression in roots. The expression trend of *IcMYB4-3* was consistent with that of *IcMYB4-2*. Among the three copies of *I*. *chlorosepala*, *IcMYB4-1* has the highest expression in the root, followed by *IcMYB4-2* and *IcMYB4-3*, indicating that *IcMYB4-1* exerts a more dominant function within the root of *I*. *chlorosepala*. Furthermore, *IcMYB4-2* has the highest expression level in the leaves, which suggests that *IcMYB4-2* primarily functions in the leaves of *I*. *chlorosepala*. Lastly, *IcMYB4-3* demonstrates the highest expression in the stem compared to the other two copies, indicating its main function in the stem of *I*. *chlorosepala*. In three different parts of *I*. *chlorosepala*, the lignification degree of roots is relatively high, with more lignin, but less lignin in leaves. It is speculated that *IcMYB4-1* gene may positively regulate lignin synthesis in *I*. *chlorosepala*, while *IcMYB4-2* and *IcMYB4-3* may have a negative effect on lignin synthesis. The study indicates that there were some differences in the mechanism of different copies of *IcMYB4* genes on lignin synthesis regulation in different parts of *I*. *chlorosepala*.

Three *IuMYB4* genes were expressed in the roots, stems, and leaves of *I. uliginosa*. The expression patterns of *IuMYB4-1*, *IuMYB4-2* and *IuMYB4-3* genes were consistent across different parts of *I. uliginosa*, with the lowest expression in the stems, the second in the roots and the highest expression in the leaves. It is speculated that all three copies have an important function in the leaves of *I. uliginosa* (Fig. 4B). The expression level of *IuMYB4-2* is the lowest among the three copies, indicating that it may have the least significant role in *I. uliginosa*. The expression level of *IuMYB4-3* in three different parts of *I. uliginosa* is highest among the three copies, and it is speculated that *IuMYB4-3* plays the strongest role in *I. uliginosa*. Three copies of *IuMYB4* of *I. uliginosa* are all the highest in the leaves, which contain the least lignin content. Despite the differences in their possible functions in various parts, they all play a similar role and negatively regulate lignin synthesis in *I. uliginosa*.

The expression of *IrMYB4-1* in the root of *I. rubrostriata* was the lowest, followed by the stem, and the highest expression was in the leaves, indicating that *IrMYB4-1* played a stronger role in the leaves of *I. rubrostriata*. *IrMYB4-2* displayed the lowest expression level in stems and the highest in leaves, implying that both *IrMYB4-1* and *IrMYB4-2* have great influence on lignin synthesis in leaves of *I. rubrostriata* (Fig. 4C). The expression levels of *IrMYB4-1* in three different parts of *I. rubrostriata* were lower than that of *IrMYB4-2*, which indicated that *IrMYB4-2* plays a stronger role in *I. rubrostriata*. The expression level of *IrMYB4* in the root and stem of *I. rubrostriata* with high lignification is low, but it is high in the leaves with low lignification, which indicates that two copies of *IrMYB4* could be involved in negative regulation of lignin synthesis in *I. rubrostriata*. However, there may be some differences in both the intensity and mechanism between the different copies.

### Expression analysis of structural genes related to lignin biosynthesis in three *Impatiens* species

**Fig. 5:**
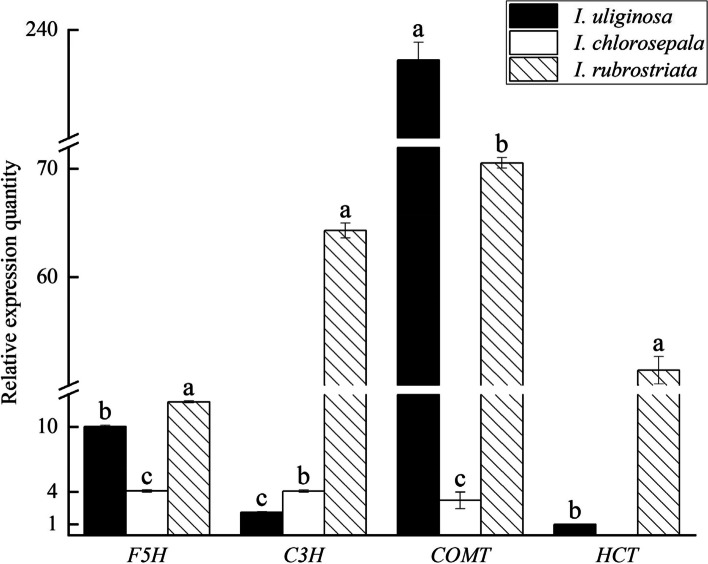
Relative expression of structural genes in the stems of three *Impatiens* species. Different lowercase letters indicate significant differences (*P* < 0.05)

Structural genes involved in lignin synthesis, such as *F5H*, *C3H*, *COMT*, and *HCT*, were all expressed in the stems of three *Impatiens* species (Fig. 5). Notably, *F5H* expression was highest in *I*. *rubrostriata*, followed by *I. uliginosa*, while *I. chlorosepala* had the lowest expression, suggesting that *F5H* has a greater effect on *I*. *rubrostriata*. The expression of *C3H* was highest in *I*. *rubrostriata*, followed by *I. chlorosepala*, and lowest in *I. uliginosa*, indicating a major role of *C3H* in the stems of *I*. *rubrostriata*. In contrast, the expression of *COMT* was the lowest in the stems of *I. chlorosepala*, followed by *I*. *rubrostriata*, and the highest in *I. uliginosa*, indicating that *COMT* may play a stronger role in the stems of *I. uliginosa*. *HCT* expression is absent in *I. chlorosepala*, which means that it does not affect on the stem of *I. chlorosepala*, and its expression in *I*. *rubrostriata* is higher than that in *I. uliginosa*, so *HCT* mainly plays a functional role in *I*. *rubrostriata*.

The stem lignin content of *I*. *rubrostriata* is the highest among the three *Impatiens* species, followed by *I. uliginosa*, with *I. chlorosepala* having the lowest content. The *F5H*, *C3H*, and *HCT* genes were most expressed in *I*. *rubrostriata*’s stems, while *COMT* was most expressed in the stems of *I. uliginosa*. *F5H*, *HCT*, and *COMT* were least expressed in *I. chlorosepala*’s stems, with *C3H* being least expressed in *I. uliginosa*’s stems. The expression levels of the aforementioned structural genes are lower in *I. chlorosepala*, but high in *I*. *rubrostriata*, which is contrary to the expression trend of *MYB4* gene in three *Impatiens* species. It can be seen that *MYB4* genes may inhibit the expression of *F5H*, *HCT*, and *COMT* in *I. chlorosepala*, resulting in the lowest lignin content in its stems and the absence of S-lignin. However, in the *I*. *rubrostriata* stem, the expression of *MYB4* gene is the lowest, resulting in the weakest inhibition on its structural gene and it may only a partial effect on *COMT*, so its lignin content is the highest. These results suggest that transcription factors such as MYB4 may regulate structural genes such as *F5H*, *C3H*, *COMT*, and *HCT*, and have certain influence on the lignin content of *Impatiens* species’ stems.

## Discussion

### The lignification degree of stems of three *Impatiens* species may be closely related to their growing environment and morphology

During plant evolution, they adapt to the environment and some physiological changes through a series of morphological and structural alterations. For instance, the anatomical structure changes of stems can enhance their ability to tolerate cold, resist drought and lodging, as well as determine their suitability for the aquatic or terrestrial environment [43].

The stems of three *Impatiens* species are composed of several layers, including the epidermis, cortex, phloem, cambium, xylem, and pith. Studies have demonstrated that plants with more developed pith and an increased number of vessels possess a greater capacity to store water [44, 45]. Among the three *Impatiens* species, *I. chlorosepala* predominantly grows in wet forest areas along streams, and the pith cells are disordered and loosely arranged. *I. uliginosa* mostly grows in water and has a hollow pit. *I*. *rubrostriata* thrives in dry hillside woodland, with closely arranged and well-developed pith cells. *I*. *rubrostriata* has more vessels compared to *I. uliginosa*, while *I. chlorosepala* has the least number of vessels. We speculate that *I*. *rubrostriata*, which grows in arid hillside woodland, may require a greater water supply during growth, which leads to more developed stem pith and more vessels.

The stem diameter of *I. uliginosa* is the largest, and the thickness of cortical cells, vessel diameter, pith diameter, xylem thickness, and phloem thickness are all larger than those of the other two *Impatiens* species. Previous studies have shown that the thickness of cortical cells, vessel diameter, xylem thickness and phloem thickness can affect a plant's water transport and energy storage of plants [46, 47]. The above indexes of *I. uliginosa* are higher than those of *I*. *rubrostriata* grown in arid areas, possibly due to differences in the shape and growth speed of the plant itself. *I. uliginosa* can grow up to 1 m per year, whereas *I*. *rubrostriata* reaches only around 0.5 m. Among the three *Impatiens* species, the cell wall of *I. uliginosa* is the thickest, while that of *I. chlorosepala* has nearly negligible cell wall. This difference may be attributed to the larger plant height and stem thickness of *I. uliginosa* as compared to *I. chlorosepala* and *I*. *rubrostriata* during the same growth period. The ratio of xylem area to stem cross-sectional area is the highest in *I*. *rubrostriata*, reaching 11.4%, *I. uliginosa* follows with 6.58%, and *I. chlorosepala* with 5.48%, which is consistent with the research results of Chen [48] the higher the ratio of xylem area to stem cross-sectional area, the higher the lignification degree of stems.

According to the aforementioned findings, one can deduce that the lignification degree of plants may be related to their growing environment and morphology. The stems of *I. chlorosepala* grow in a creeping manner, resulting in weak mechanical support, thus leading to a thin cell wall and xylem and a low the ratio of xylem area to stem cross-sectional area. The degree of lignification in *I. uliginosa* with erect stems is higher than that of *I. chlorosepala* with creeping stems. However, the lignification degree of *I*. *rubrostriata* with the same upright stem is greater than that of *I. uliginosa* with upright stem, suggesting a potential correlation between lignification and plant growth environment. Plants in an aquatic environment exhibit a lower degree of lignification, whereas those in a terrestrial environment exhibit a higher degree. For example, Wang's [49] research found that *Alternanthera philoxeroides* exhibits higher lignification levels in terrestrial environments compared to aquatic habitats. Nevertheless, the lignification degree of plants is not only related to the morphological anatomy of stems, but also related to the lignin content in stems. Thus, a comprehensive examination of stem lignification from various perspectives is necessary.

### Comparative analysis of lignin content and composition in stems of three *Impatiens* species

To support our results on stem morphology, we analyzed the lignin content and lignin monomer content in three *Impatiens* species. Lignin is a secondary metabolite that plays a crucial role in plant growth, development, and response to adverse environmental stress. The concentration of lignin varies not only between different species but also within the same species at different growth stages. Typically, coniferous wood has a lignin content of 27–33%, while broadleaf wood and herbs have 18–25% and 17–24%, respectively [50]. There is a positive correlation between the total lignin determination and the morphological anatomy of the stem. The total lignin content of *I. chlorosepala* is 4.21%, which differs significantly from *I. uliginosa* and *I*. *rubrostriata*. The variation in total lignin content among three *Impatiens* species may be connected to their upright or creeping growth habits.

Studies have shown that S-lignin is related to the mechanical strength and vertical growth of plants [51]. In the determination of lignin monomer content, *I. chlorosepala* lacks S-lignin, and there is no significant difference in S-lignin between *I. uliginosa* and *I*. *rubrostriata*, which is consistent with the morphological characteristics of the creeping growth of stems of *I. chlorosepala* and the vertical growth of *I. uliginosa* and *I*. *rubrostriata*. The H-lignin and G-lignin of *I*. *rubrostriata* were significantly higher than those of *I. uliginosa*, while *I. chlorosepala* showed the lowest value. This finding provides evidence that the lignification degree of *I*. *rubrostriata* with upright stems growing in arid hillside woodlands is the highest, and that of *I. uliginosa* with erect stems in water is higher than that of *I. chlorosepala* with creeping stems in wet forests. The results show that the lignification degree of plant stems may be closely related to their growth characteristics and lignin content.

### The regulatory role of *MYB4* gene in lignin biosynthesis of three *Impatiens* species

The *MYB4* genes from three *Impatiens* species that we isolated and cloned belong to the MYB family. Many researches show that theR2R3-MYB family has the N-terminal amino acid motif that is relatively more conservative and the C-terminal amino acid motif that is extremely low [52, 53]. It is consistent with the results of this study, the DNA domain of MYB transcription factors is highly conserved across species. Phylogenetic analysis indicates that the MYB4 genes in three *Impatiens* species share a close evolutionary relationship with plants including *Camellia sinensis*, *Actinidia rufa* and *Vitis vinifera*. *CsMYB4* in *Camellia sinensis* [54], and *VvMYB4b* in *Vitis vinifera* ‘Moldova’ [55], have both been shown to inhibit lignin synthesis, and it is possible that the *MYB4* genes in three *Impatiens* species also play a negative regulatory role in lignin synthesis. The *MYB4* gene’s copy number varies among plant species, and they are expressed in all parts of most plants. Even within the same species, the expression level significantly varies among different plant parts. For instance, *CsMYB4-5* and *CsMYB4-6* exhibit high expression in roots and low expression in stems in *Camellia sinensis* [54]. In this study, the expression levels of *MYB4* genes were lower in roots or stems and higher in leaves of three *Impatiens* species. This trend is consistent with the observed expression pattern observed in other plants. We speculated that the MYB4 transcription factors may have transcriptional inhibition.

Research has demonstrated that overexpression of the *TaMYB4* transcription factor in wheat leads to a decrease in total lignin content [56]. In *Arabidopsis*, *MYB4* suppresses the expression of genes related to lignin [18]. The studies demonstrate that the *MYB4* gene inhibits the regulation of lignin synthesis. However, the overexpression of the *PbrMYB4* gene in pear calli and *Arabidopsis thaliana* promotes lignin accumulation in *Pyrus bretschneideri* fruit stone cells. This provides evidence that the *PbrMYB4* gene has a positive regulatory effect on lignin biosynthesis in pear fruit stone cells [57]. It indicates that the regulation of the *MYB4* gene on lignin may vary among different plants. In this study, it was also found that *IcMYB4-1* exhibited the highest expression in the root of *I. chlorosepala*, while having low expression in the stem and leaf, suggesting that it may exert a positive influence on regulating lignin synthesis in *I. chlorosepala*. The two other *IcMYB4* genes show the highest expression levels in leaves, but the expression levels are relatively low in roots and stems with high lignification, so it is speculated that they may play a negative regulatory role in lignin synthesis of *I. chlorosepala*. The expression levels of *MYB4* of *I. uliginosa* and *I*. *rubrostriata* are low in roots and stems with high lignin content, and high in leaves with low lignin content. Further verification is needed to determine the regulatory mechanism and function of the *MYB4* genes in the regulation of lignin synthesis in *Impatiens* species, as indicated by the aforementioned findings.

The expansion and contraction of genes play a crucial role in the adaptive evolution of plants [58, 59]. For example, in four Poaceae species (rice, maize, sorghum, and brachypodium), NBS genes showed a contraction evolutionary pattern [58, 60]. In the study on Orchidaceae, Wang et al. [61] found that CslA, a gene related to plant cell wall polysaccharide synthesis with expansion, may play a key role in the response of different life forms of Orchidaceae (epiphytic, terrestrial, and saprophytic) to drought stress. This study found that aquatic *I. uliginosa* and wet *I. chlorosepala* have three *MYB4* genes, while terrestrial *I. rubrostriata* has only two *MYB4* genes. Whether this change in the number of *MYB4* genes between *Impatiens* species is related to plant adaptation to the environment and/or the growth state of the plants themselves. It is also unclear whether the gene expansion from terrestrial to aquatic environments or gene contraction from aquatic to terrestrial environments is responsible. Additionally, while *MYB4* negatively regulates lignin biosynthesis, but the expression of *IcMYB4-1* in *I. chlorosepala* is positively correlated with lignin content. Further study is required to determine whether gene expansion during adaptive evolution leads to the functionalization of genes.

### *MYB4* negatively regulates lignin biosynthesis by inhibiting the expression of structural genes

*MYB4* regulates lignin synthesis by controlling the expression of structural genes in lignin synthesis pathway. Yu [24] found that lignin-related genes *CCR* and *C4H* were down-regulated in birch that overexpressed *BpMYB4*, *CAD* and *C4H* genes were up-regulated in birch which suppressed *BpMYB4*, which indicates that BpMYB4 transcription factor can negatively affect the expression of some lignin biosynthesis genes compared to wild type. When *PvMYB4* was overexpressed in *Panicum virgatum*, the expression levels of structural genes in transgenic plants decreased obviously, including *COMT*, *C3H*, *F5H*, and *HCT* [22]. This research shows that *MYB4* has the ability to inhibit the expression of structural genes. In this study, the expression levels of structural genes *F5H* and *HCT* were the lowest in *I. chlorosepala* and the highest in *I*. *rubrostriata*, which contradicts the expression trend of *MYB4* in the stems of three *Impatiens* species*.* It was speculated that *MYB4* could inhibit the expression of the two structural genes. Additionally, *MYB4* expression was the highest in the stem of *I. chlorosepala*, resulting in the lowest expression of *C3H* and *COMT* among the three *Impatiens* species. The expression levels of *C3H* and *COMT* are high in *I*. *rubrostriata*. It is hypothesized that the low expression level of *MYB4* in *I*. *rubrostriata* leads to a feeble inhibitory effect on the two structural genes.

The synthesis process of lignin monomers can be regulated by reducing the activity of structural genes. Overexpression of the F5H structural gene in plants results in the production of a large quantity of S-lignin in the plants [62, 63]. *COMT* regulates G-lignin, but has the capability of also regulating S-lignin [64]. *C3H* and *HCT* heavily influence H-lignin [65, 66]. *I. chlorosepala* lacks S-lignin, so it is consistent with the lowest expression level of *F5H* in the stems of *I. chlorosepala*. Moreover, the H-and G-lignin of *I. chlorosepala* are lower than these of *I. uliginosa* and *I*. *rubrostriata*, there is no *HCT* expression in *I. chlorosepala*, but the expression of *C3H* is higher than that of *I. uliginosa*. It is speculated that the H-lignin of *I. chlorosepala* is mainly generated by *C3H* catalysis. The three lignin monomers of *I. uliginosa* are all lower than those of *I*. *rubrostriata*. Additionally, the expression of other structural genes, except for *COMT*, is higher in *I*. *rubrostriata*, which may be attributed to the fact that *COMT* is also involved in the synthesis of S-lignin in *I. uliginosa*. Among the three *Impatiens* species, *I*. *rubrostriata* exhibits the highest content of three lignin monomers, and the expression of its structural genes positively correlates with the lignin monomer content. The synthesis of lignin monomer is supposed to be controlled by the structural genes in the stems of three *Impatiens* plants regulated by MYB4 transcription factors, thus affecting the lignin content and the lignification degree of *Impatiens* species. However, the lignin synthesis pathway is completed through multi-gene and multi-level coordination; therefore, further exploration is necessary to understand the specific pathway of lignin synthesis in *Impatiens*.

## Conclusion

There are significant differences in anatomical parameters among the stems of three *Impatiens* species. Moreover, the stems of *I. rubrostriata* exhibit the highest content of lignin and lignin monomer, followed by *I. uliginosa* and *I. chlorosepala*. The anatomical structure is consistent with the determination results of lignin content. *I*. *rubrostriata* has a higher degree of lignification due to its upright and solid stems, while *I. uliginosa* exhibits a lower degree of lignification with its upright and hollow stems, and *I. chlorosepala* displays the lowest degree of lignification owing to its creep and solid stems.

Three copies of *MYB4* genes in *I. uliginosa* and *I. chlorosepala* and two copies of *I*. *rubrostriata* were isolated and cloned. The MYB4 proteins in three *Impatiens* species demonstrate high similarity with other plants. They possess conserved R2 and R3 DNA binding domains at the N-terminal and demonstrate a high homology. In addition, they have an inhibitory conserved motif EAR at the C-terminal. Except for *IcMYB4-1*, which was the highest expression level in the root of *I. chlorosepala*, and the other *MYB4* genes of three *Impatiens* species were mainly expressed in the leaves, with the stems and roots following behind. This suggests the potential for disparities between the different copies of the *MYB4* gene within the three *Impatiens* species. According to the lignin content and the differential expression of *MYB4* and structural genes in the stems of three *Impatiens* species, it is speculated that *MYB4* negatively regulates the lignin synthesis in the stems of three *Impatiens* species by regulating the expression of structural genes, and its regulation mechanism appears to vary across different *Impatiens* species.

### Supplementary Information

Supplementary Material 1.

## Data Availability

Sequences of MYB4 genes were obtained from three *Impatiens *species, all sequences have been uploaded to the NCBI GeneBank with the following accession number: PP426043-PP426050 (https://www.ncbi.nlm.nih.gov/nuccore/PP426043/ to https://www.ncbi.nlm.nih.gov/nuccore/PP426050/).
