# Supplementary material for: Adaptation mechanism of three Impatiens species to different habitats based on stem morphology, lignin and MYB4 gene
Source: BMC Plant Biol. 2024 May 24;24:453. doi: 10.1186/s12870-024-05115-3 (PMC11127381; doi:10.1186/s12870-024-05115-3)
Supplement: Supplementary file 1 — Supplementary Material 1. [file 12870_2024_5115_MOESM1_ESM.doc]

Table S1 Observation index of paraffin sections of stems from *Impatiens*.

| **Plant organ** | **Stems** | | |
| --- | --- | --- | --- |
| **Index** | epidermis (Ep) | | |
|  | cortex (Co) | | |
|  | stem diameter (D) | | |
|  | vascular bundle (Vb) | | |
|  | Vessel (Ve) | | |
|  | cambium (Vc) | | |
|  | xylem (Xyl) | | |
|  | phloem (Ph) | | |
|  | cell wall (Cw) | | |
|  | xylem area (Sxyl) | | |
|  | ratio of vascular bundle area to stem cross-sectional area (Vb/S)= | Vb | ×100% |
|  |  | S |  |
|  | ratio of cell wall thickness to stem diameter (Cw/D)= | Cw | ×100% |
|  |  | D |  |
|  | ratio of xylem area to stem cross-sectional area (Sxyl/S)= | Sxyl | ×100% |
|  |  | S |  |
|  | ratio of xylem thickness to stem diameter (Xyl/D)= | Xyl | ×100% |
|  |  | D |  |

Table S2 Primers for the cDNA cloning of *MYB*4 in three *Impatiens*.

| **Primer name** | **Primer sequence 5’-3’** |
| --- | --- |
| *IcMYB*4-1.F | ATGGGAAGGTCTCCCTG |
| *IcMYB*4-1.R | TCATTTCATCTCCAATGTTCTGTAATCG |
| *IcMYB*4-2.F | ATGGGAAGGTCTCCTTGC |
| *IcMYB*4-2.R | TCAGATCTTGAATTTCATCTCCAAGTTTC |
| *IcMYB*4-3.F | ATGGGAAGGTCTCCTTGC |
| *IcMYB*4-3.R | CTAGAACTTCATCTCCAAGCTTCTG |
| *IuMYB*4-1.F | ATGGGAAGGTCTCCCTG |
| *IuMYB*4-1.R | TCATTTCATCTCCAAGCCTCTG |
| *IuMYB*4-2.F | ATGAGGAATCCTTGTTGCGATAAAC |
| *IuMYB*4-2.R | TTAACTAAAAAGTGGAAGGGTAGGAGG |
| *IuMYB*4-3.F | ATGAGAATCATGAGAAACCCTTGC |
| *IuMYB*4-3.R | TTACGTGAAGAGGGCGAG |
| *IrMYB*4-1.F | ATGGGAAGGTCTCCCTG |
| *IrMYB*4-1.R | TCATTTCATCTCCAAACTTCTATAATCG |
| *IrMYB*4-2.F | ATGGGAAGGTCTCCTTGCTG |
| *IrMYB*4-2.R | TCAGATCTTGAACTTCATCTCCAAGC |

Table S3 Primers in *Impatiens* for qRT PCR

| **Primer name** | **Primer sequence 5’-3’** |
| --- | --- |
| *IcActin*.F | CTTGATTTGGCTGGTCGGGA |
| *IcActin*.R | TTGACGGCTGGAACAAGACC |
| *IuActin*.F | TGAATGTCCCTGCTGTTTG |
| *IuActin*.R | ACCTTCCGCATAACTTTACC |
| *IrActin*.F | GCGGGATCTGACTGACCATT |
| *IrActin*.R | GACCTCGGGGCATCTAAACC |
| *A.*F | GTTCTGTCCATTCCCATCTGTC |
| *A.*R | CCCTGAGGAATCCAGTGAGC |
| *IcMYB*4-1.F | CGGATGAGAGATGCCCTGAC |
| *IcMYB*4-1.R | GCGGCTGATCTCAACCCTAA |
| *IcMYB*4-2.F | GCGTCCTAGGCAACAAATGG |
| *IcMYB*4-2.R | GGGCTCATACTCAGCTCCAG |
| *IcMYB*4-3.F | ATGGTCGTTAATCGCGGGAA |
| *IcMYB*4-3.R | TCGAGGTTCAAATCAGGGCA |
| *IuMYB*4-1.F | ACAAATGGTCGCTGATTGCC |
| *IuMYB*4-1.R | TCAAGGGATTGAGCCGGAAG |
| *IuMYB*4-2.F | ATTCAAAGACACGGCGAAGG |
| *IuMYB*4-2.R | GGTTCTTCCAGGCAGTCGTC |
| *IuMYB*4-3.F | GCCCGTTCCTGAATCAAACG |
| *IuMYB*4-3.R | GGTCCTTTCTTTTGCGACGG |
| *IrMYB*4-1.F | TCGACTCATCGCTTACATCCG |
| *IrMYB*4-1.R | CGTTCTACCCGGCATCCTTC |
| *IrMYB*4-2.F | CGGGGAGAACAGACAACGAG |
| *IrMYB*4-2.R | GTGGTAGTGGTGGTGGTCAA |
| *MYB*4.F | CTCGGCAACAAATGGTCG |
| *MYB*4.R | CGGGCTGATTCTMAGCTC |
| *COMT*.F | TCATGGAAAGCTGGTACC |
| *COMT*.R | AACATGAGGCAAATCAAA |
| *F5H*.F | ACCCGGATATGGTGGATGAC |
| *F5H*.R | GGGCTTTTCAWCAGCTCCG |
| *C3H*.F | GCCATGGTTGAATCCATCTTC |
| *C3H*.R | GAACATCCATCTTAGCCAAGG |
| *HCT*.F | CCATTTCGTCAACACATGGTC |
| *HCT*.R | GTGCAACGCCAGATGTG |

*Table S4 Correlation anatomical structure of I. chlorosepala*

| **Index** | **D** | **Xyl** | **Cw** | **Sxyl** | **Vb** | **Cw/ D** | **Xyl/D** | **Vb/S** | **Sxyl / S** |
| --- | --- | --- | --- | --- | --- | --- | --- | --- | --- |
| **D** | 1 |  |  |  |  |  |  |  |  |
| **Xyl** | -0.305 | 1 |  |  |  |  |  |  |  |
| **Cw** | 0.408 | -0.251 | 1 |  |  |  |  |  |  |
| **Sxyl** | 0.432 | 0.652 | -0.017 | 1 |  |  |  |  |  |
| **Vb** | -0.237 | 0.719 | 0.01 | 0.672 | 1 |  |  |  |  |
| **Cw/D** | -0.075 | -0.117 | 0.880^*^ | -0.248 | 0.129 | 1 |  |  |  |
| **Xyl/D** | -0.633 | 0.916^*^ | -0.438 | 0.314 | 0.564 | -0.151 | 1 |  |  |
| **Vb/S** | -0.594 | 0.717 | -0.146 | 0.383 | 0.922^*^ | 0.145 | 0.718 | 1 |  |
| **Sxyl/S** | -0.163 | 0.912^*^ | -0.268 | 0.819 | 0.885^*^ | -0.213 | 0.750 | 0.796 | 1 |

^*^ means *P*<0.05.

*Table S5 Correlation anatomical structure of I. uliginosa*

| **Index** | **D** | **Xyl** | **Cw** | **Sxyl** | **Vb** | **Cw/ D** | **Xyl/D** | **Vb/S** | **Sxyl / S** |
| --- | --- | --- | --- | --- | --- | --- | --- | --- | --- |
| **D** | 1 |  |  |  |  |  |  |  |  |
| **Xyl** | -0.567 | 1 |  |  |  |  |  |  |  |
| **Cw** | -0.549 | 0.221 | 1 |  |  |  |  |  |  |
| **Sxyl** | 0.527 | 0.122 | 0.198 | 1 |  |  |  |  |  |
| **Vb** | 0.407 | 0.403 | -0.613 | 0.425 | 1 |  |  |  |  |
| **Cw/D** | -0.625 | 0.279 | 0.994^**^ | 0.141 | -0.590 | 1 |  |  |  |
| **Xyl/D** | -0.769 | 0.959^**^ | 0.334 | -0.084 | 0.206 | 0.410 | 1 |  |  |
| **Vb/S** | -0.363 | 0.840 | -0.213 | 0.004 | 0.703 | -0.128 | 0.801 | 1 |  |
| **Sxyl/S** | -0.798 | 0.755 | 0.750 | 0.085 | -0.127 | 0.807 | 0.853 | 0.480 | 1 |

^**^ means *P*<0.01.

*Table S6 Correlation anatomical structure of I. rubrostriata*

| **Index** | **D** | **Xyl** | **Cw** | **Sxyl** | **Vb** | **Cw/ D** | **Xyl/D** | **Vb/S** | **Sxyl / S** |
| --- | --- | --- | --- | --- | --- | --- | --- | --- | --- |
| **D** | 1 |  |  |  |  |  |  |  |  |
| **Xyl** | 0.141 | 1 |  |  |  |  |  |  |  |
| **Cw** | 0.01 | 0.847 | 1 |  |  |  |  |  |  |
| **Sxyl** | 0.341 | 0.752 | 0.697 | 1 |  |  |  |  |  |
| **Vb** | -0.124 | 0.082 | 0.303 | 0.628 | 1 |  |  |  |  |
| **Cw/ D** | -0.147 | 0.816 | 0.987^**^ | 0.645 | 0.331 | 1 |  |  |  |
| **Xyl/D** | 0.098 | 0.999^**^ | 0.857 | 0.754 | 0.110 | 0.834 | 1 |  |  |
| **Vb/S** | -0.923^*^ | -0.056 | 0.127 | -0.03 | 0.495 | 0.275 | -0.008 | 1 |  |
| **Sxyl / S** | -0.492 | 0.574 | 0.627 | 0.650 | 0.689 | 0.706 | 0.611 | 0.720 | 1 |

^*^ means *P*<0.05, ^**^ means *P*<0.01.

Table S7 Correlation between total lignin content and anatomical structure of three *Impatiens*

| **correlation index** | **correlation coefficient** |
| --- | --- |
| xylem thickness | 0.724 |
| vascular bundle area | 0.659 |
| cell wall thickness | 0.731 |
| xylem area | 0.553 |
| ratio of vascular bundle area to stem cross-sectional area | 0.592 |
| ratio of cell wall thickness to stem diameter | 0.820 |
| ratio of xylem thickness to stem diameter | 0.660 |
| ratio of xylem area to stem cross-sectional area | 0.674 |

Table S8 Sequence-related information of *MYB4* gene in three *Impatiens*.

| **Gene name** | **CDS length** | **Number of introns** | **Intron length** | **Genome sequence** |
| --- | --- | --- | --- | --- |
| *IcMYB*4-1 | 666 | 1 | 83 | 749 |
| *IcMYB*4-2 | 888 | 1 | 107 | 995 |
| *IcMYB*4-3 | 771 | 1 | 117 | 888 |
| *IrMYB*4-1 | 660 | 1 | 82 | 742 |
| *IrMYB*4-2 | 888 | 1 | 75 | 963 |
| *IuMYB*4-1 | 642 | 1 | 91 | 733 |
| *IuMYB*4-2 | 657 | 1 | 158 | 815 |
| *IuMYB*4-3 | 639 | 2 | 70/114 | 823 |

Table S9 Physicochemical properties of MYB4 proteins from three *Impatiens* species.

| **protein** | **Total number of atoms** | **Molecular weight** | **Theoretical pI** | **Formula** | **The instability index** | **Grand average of hydropathicity** |
| --- | --- | --- | --- | --- | --- | --- |
| IcMYB4-1 | 3493 | 24984.45 Da | 8.9 | C_1086_H_1740_N_332_O_324_S_11_ | 50.19 | -0.672 |
| IcMYB4-2 | 4638 | 33110.56 Da | 6.43 | C_1434_H_2319_N_419_O_452_S_14_ | 52.27 | -0.501 |
| IcMYB4-3 | 4057 | 29165.08 Da | 9.12 | C_1259_H_2012_N_394_O_378_S_14_ | 49.79 | -0.793 |
| IuMYB4-1 | 3380 | 24153.37 Da | 8.79 | C_1054_H_1682_N_318_O_318_S_8_ | 61.15 | -0.773 |
| IuMYB4-2 | 3465 | 24762.15 Da | 8.94 | C_1070_H_1730_N_326_O_328_S_11_ | 42.7 | -0.667 |
| IuMYB4-3 | 3423 | 24335.65 Da | 8.52 | C_1067_H_1712_N_312_O_325_S_7_ | 44.68 | -0.821 |
| IrMYB4-1 | 3475 | 24772.26 Da | 8.19 | C_1084_H_1736_N_320_O_325_S_10_ | 59.14 | -0.645 |
| IrMYB4-2 | 3465 | 32969.22 Da | 8.29 | C_1425_H_2288_N_420_O_451_S_14_ | 47.66 | -0.587 |

Table S10 Subcellular localization of MYB4 protein of three *Impatiens* species

| **Gene** | **Nucleus** | **Mitochondria** | **Cytoplasmic** | **Golgi** |
| --- | --- | --- | --- | --- |
| IcMYB4-1  IcMYB4-2  IcMYB4-3 | 87.00%  60.90%  56.50% | 13.00%  13.00%  30.4% | 0.00%  21.70%  13.00% | 0.00%  4.30%  0.00% |
| IuMYB4-1  IuMYB4-2  IuMYB4-3 | 87.00%  78.30%  82.60% | 4.30%  4.30%  8.70% | 4.30%  13.00%  0.00% | 4.30%  4.30%  0.00% |
| IrMYB4-1 | 91.3% | 8.70% | 0.00% | 0.00% |
| IrMYB4-2 | 60.9% | 17.4% | 21.7% | 0.00% |


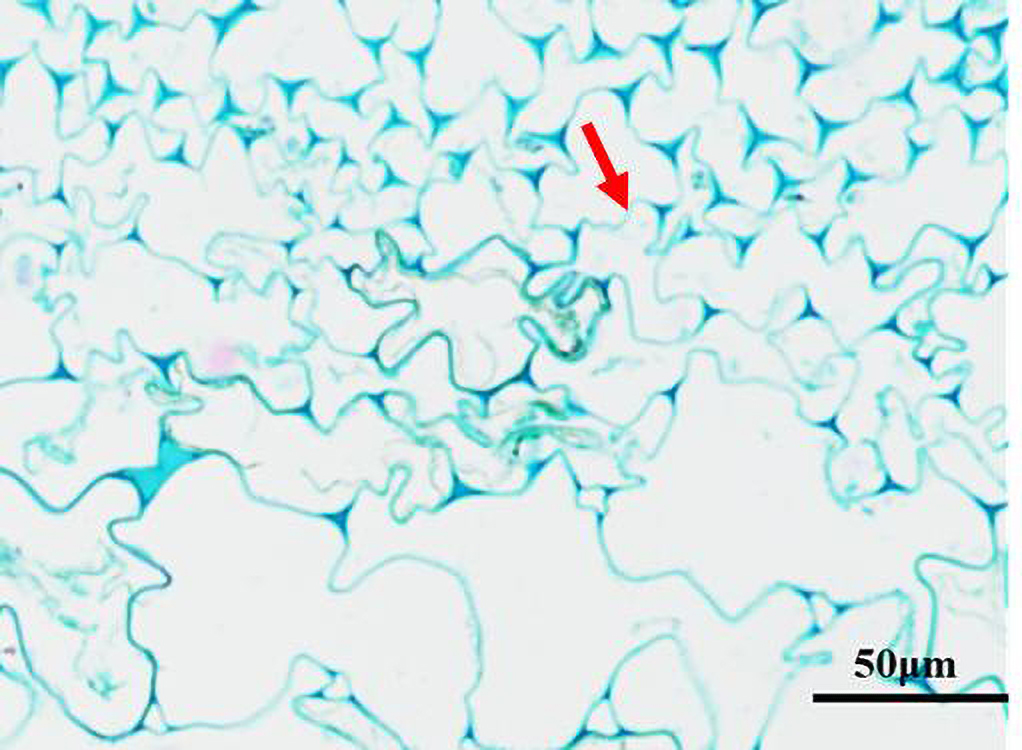

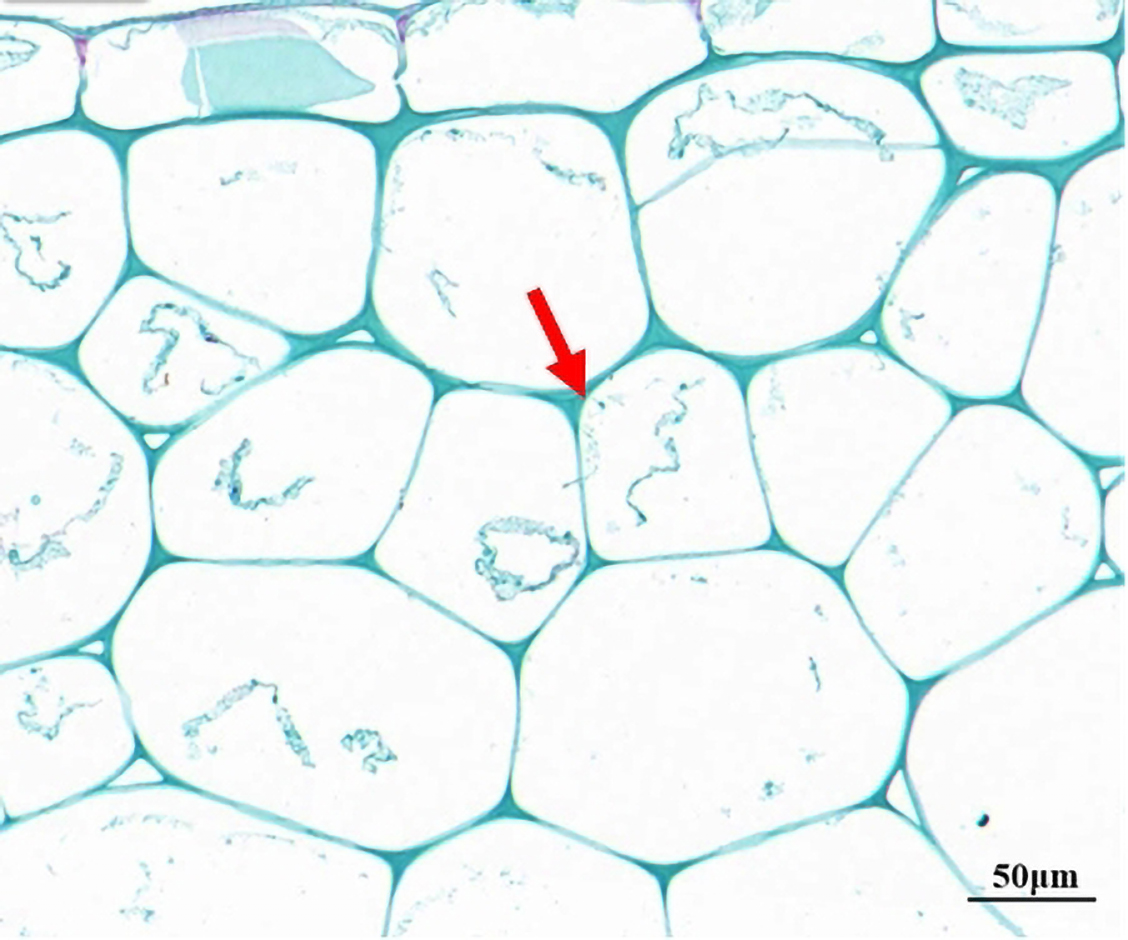

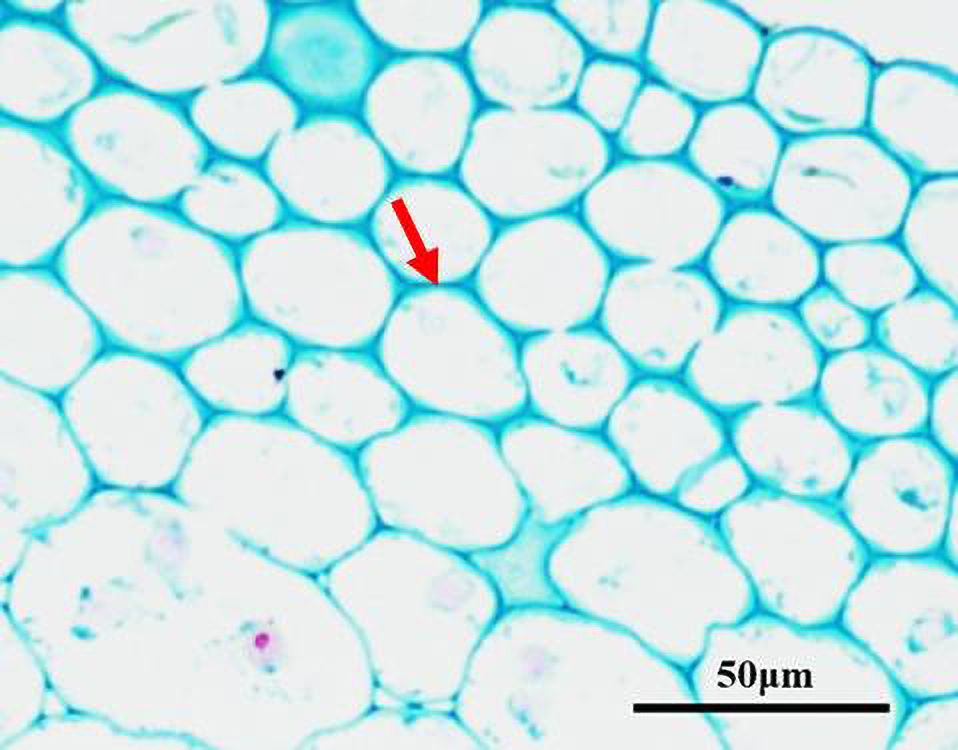


**A**

**B**

**C**

**C**

Figure S1 Cell wall of the stems of *Impatiens.* A: *I. chlorosepala*, B: *I. uliginosa*, C: *I. rubrostriata*.


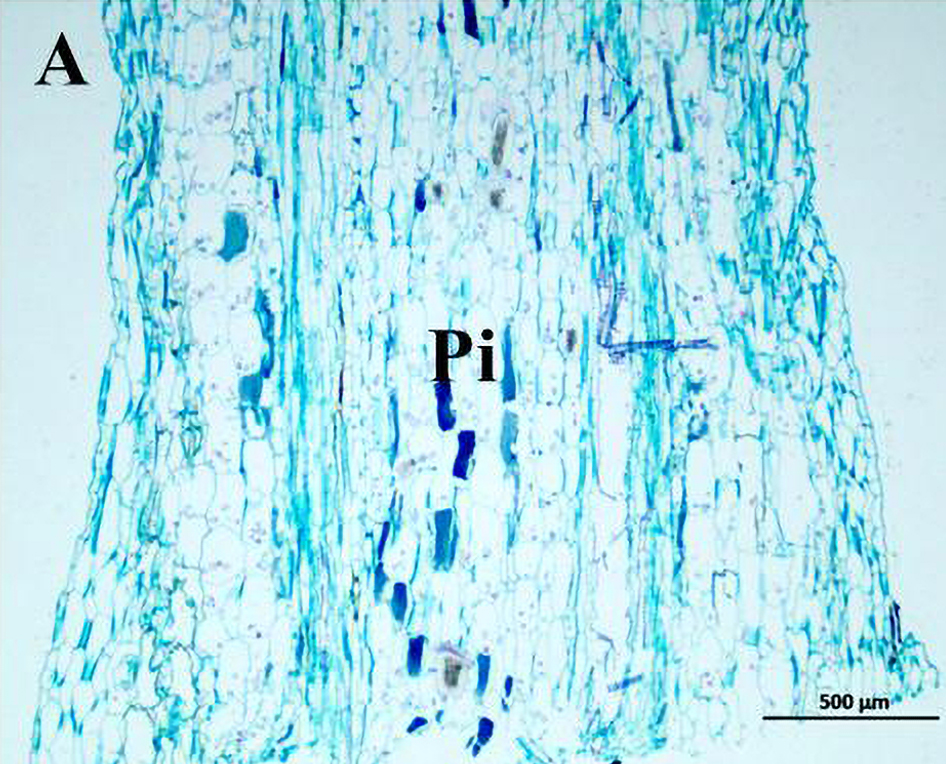

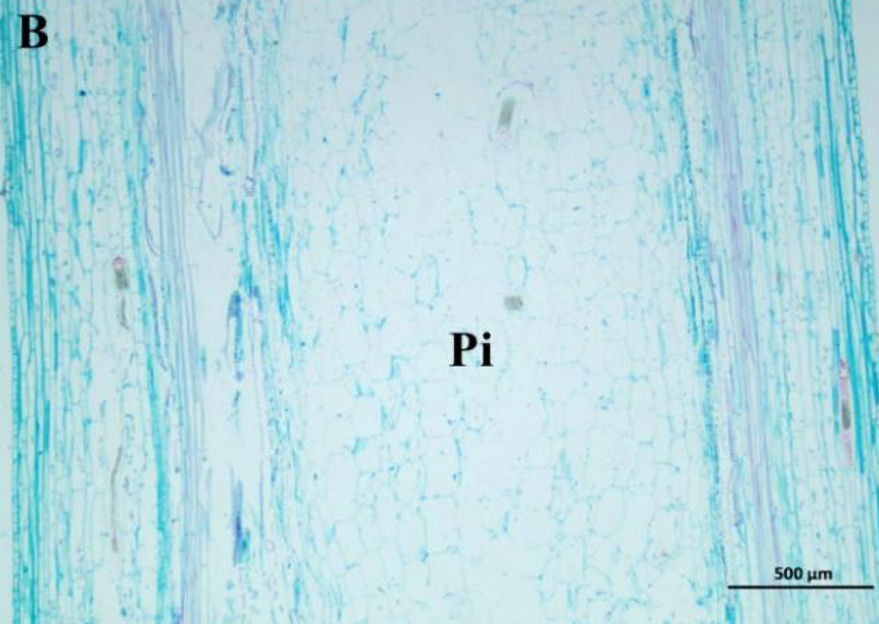

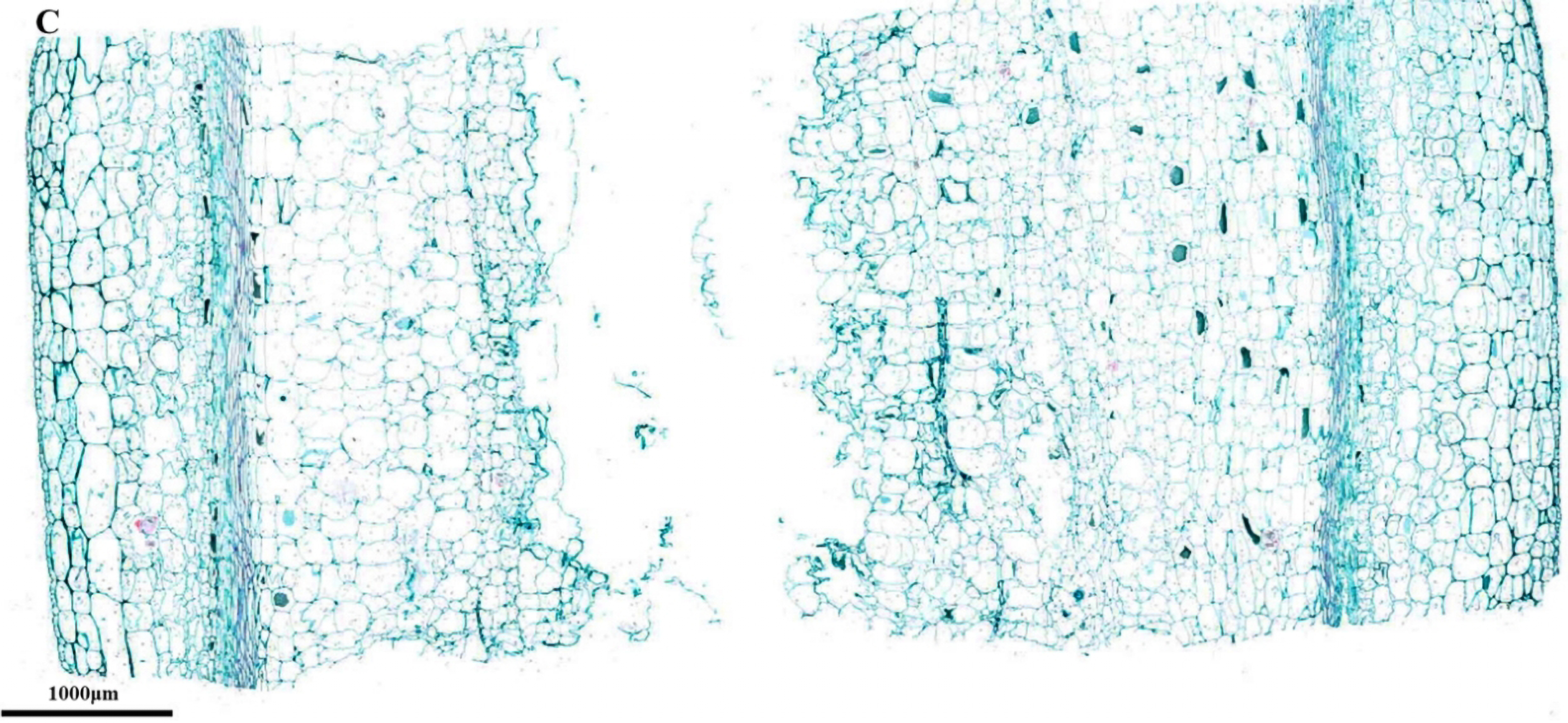


Figure S2 Longitudinal section of the stems of *Impatiens*. A: *I. chlorosepala*, B: *I. rubrostriata*, C: *I. uliginosa*.


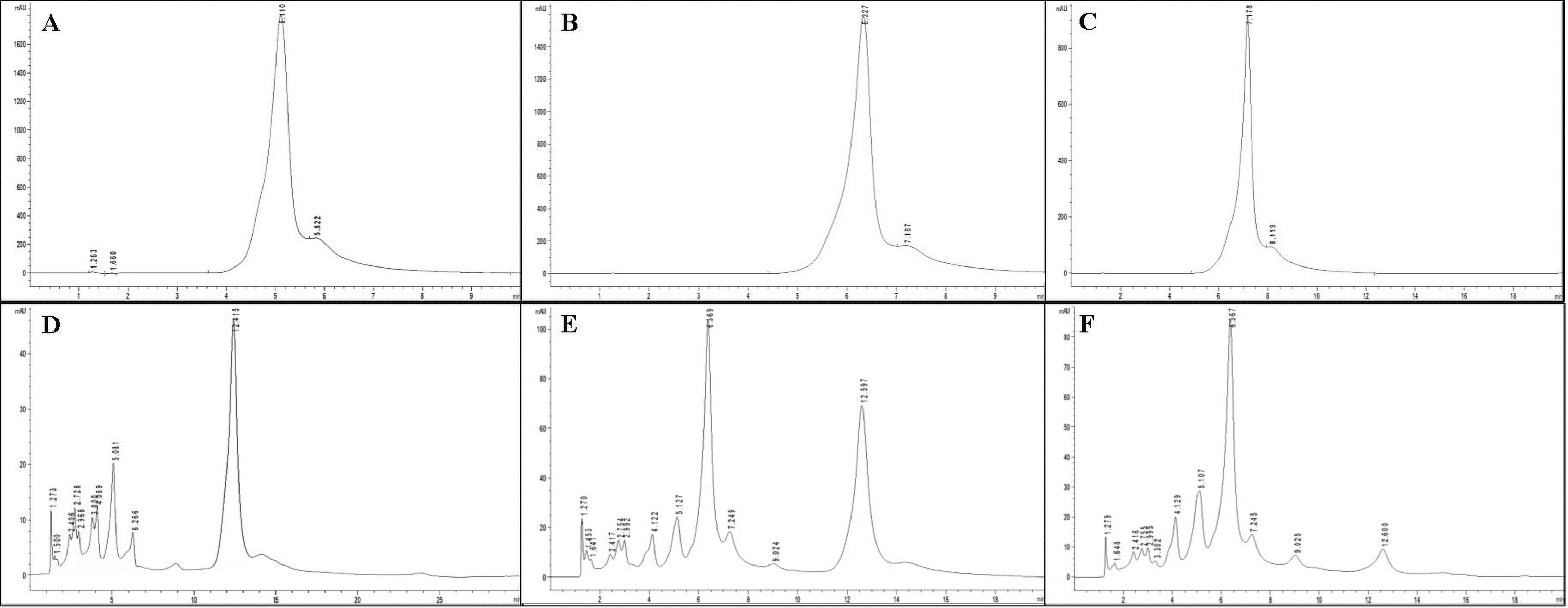
Figure S3 Peak diagram of lignin monomer standard of three *Impatiens* (A, B, C) and determination peak of lignin monomer content of *Impatiens*(D, E, F). A: 4-Hydroxybenzaldehyde (H). B: Vanillin (G). C: 3,5-Dimethoxy-4-hydroxybenzaldehyde (S). D: *I. chlorosepala.* E: *I. uliginosa*. F: *I. rubrostriata*.


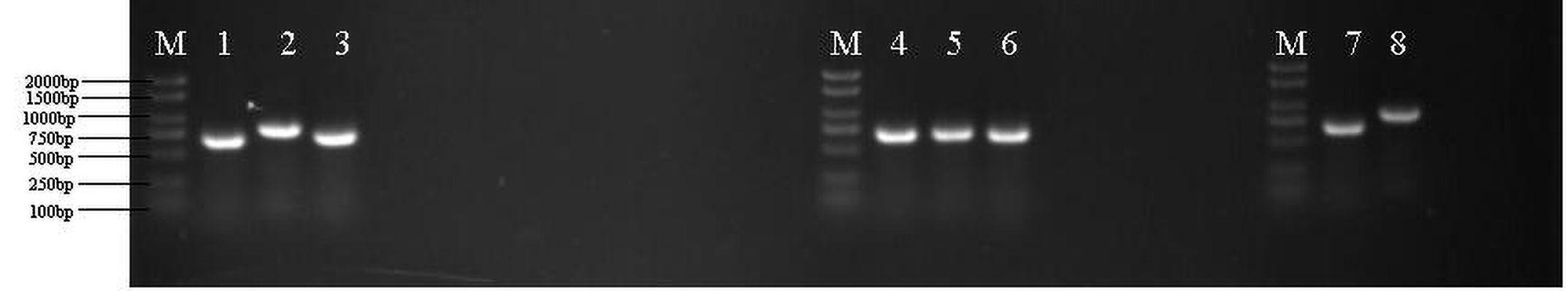


Figure S4 cDNA PCR amplification of *MYB*4

M: Marker. 1, 2, 3: *IcMYB*4-1, *IcMYB*4-2, *IcMYB*4-3. 4, 5, 6: *IuMYB*4-1, *IuMYB*4-2, *IuMYB*4-3. 7, 8: *IrMYB*4-1, *IrMYB*4-2.


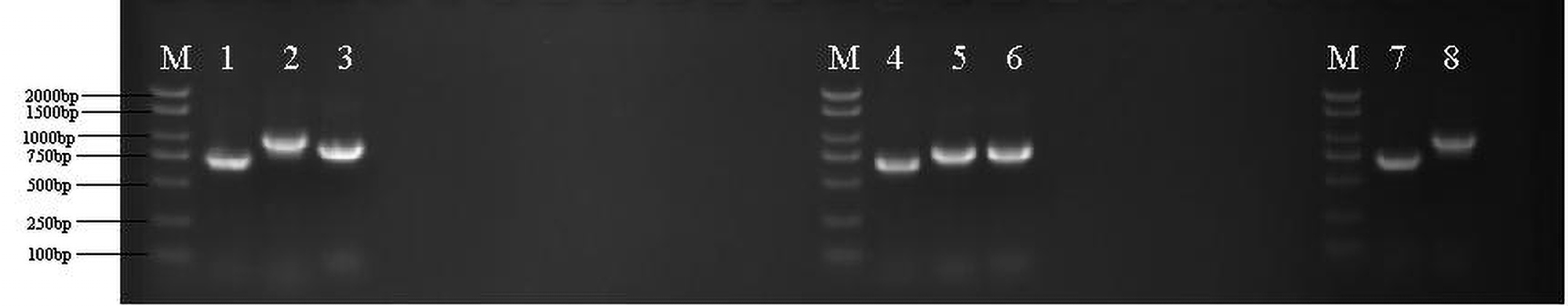


Figure S5 gDNA PCR amplification of *MYB*4

M: Marker. 1, 2, 3: *IcMYB*4-1, *IcMYB*4-2, *IcMYB*4-3. 4, 5, 6: *IuMYB*4-1, *IuMYB*4-2, *IuMYB*4-3. 7, 8: *IrMYB*4-1, *IrMYB*4-2.


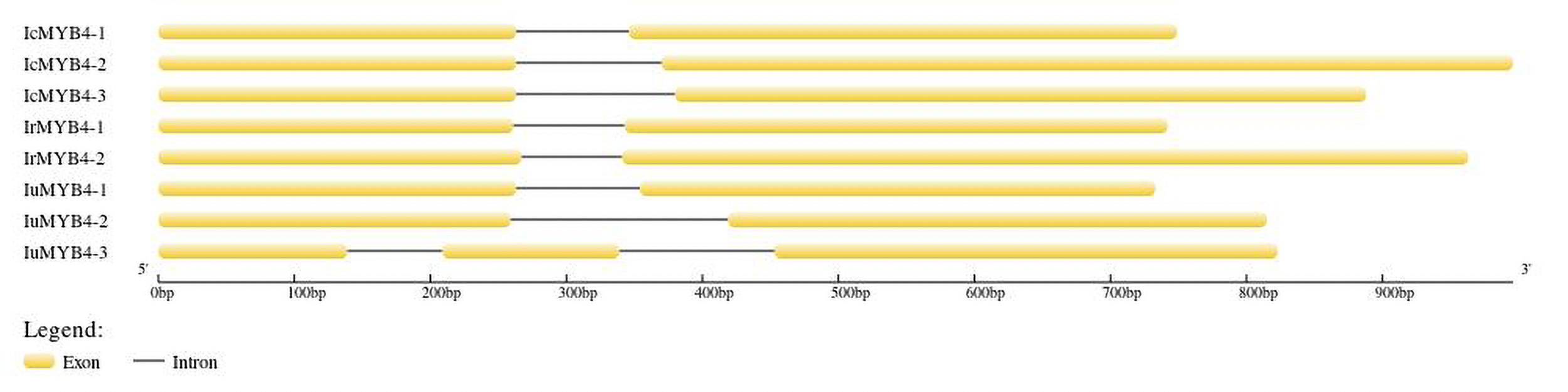


Figure S6 Six Genome Structure Map of MYB4 of three Impatiens.


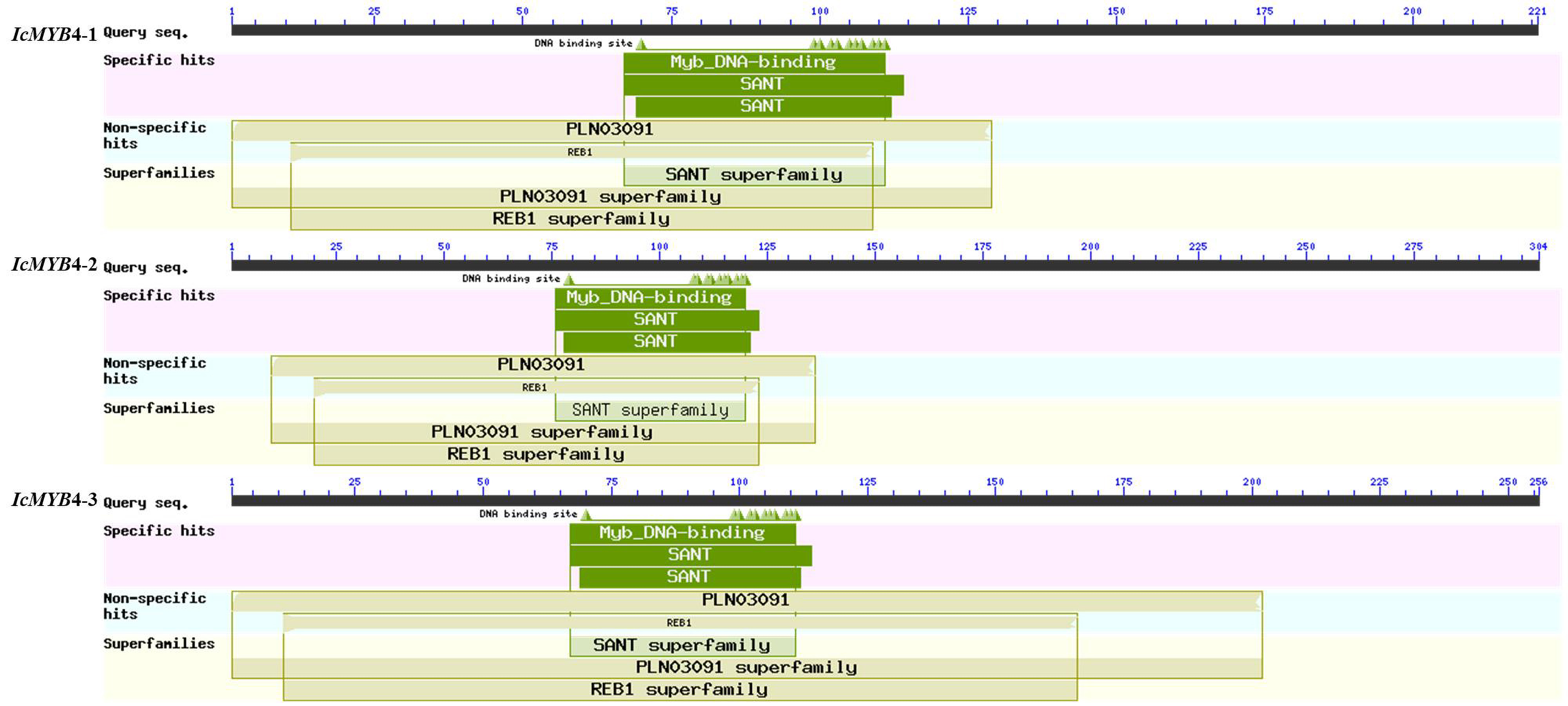


Figure S7 Conserved domains and superfamily of IcMYB4-1, IcMYB4-2 and IcMYB4-3


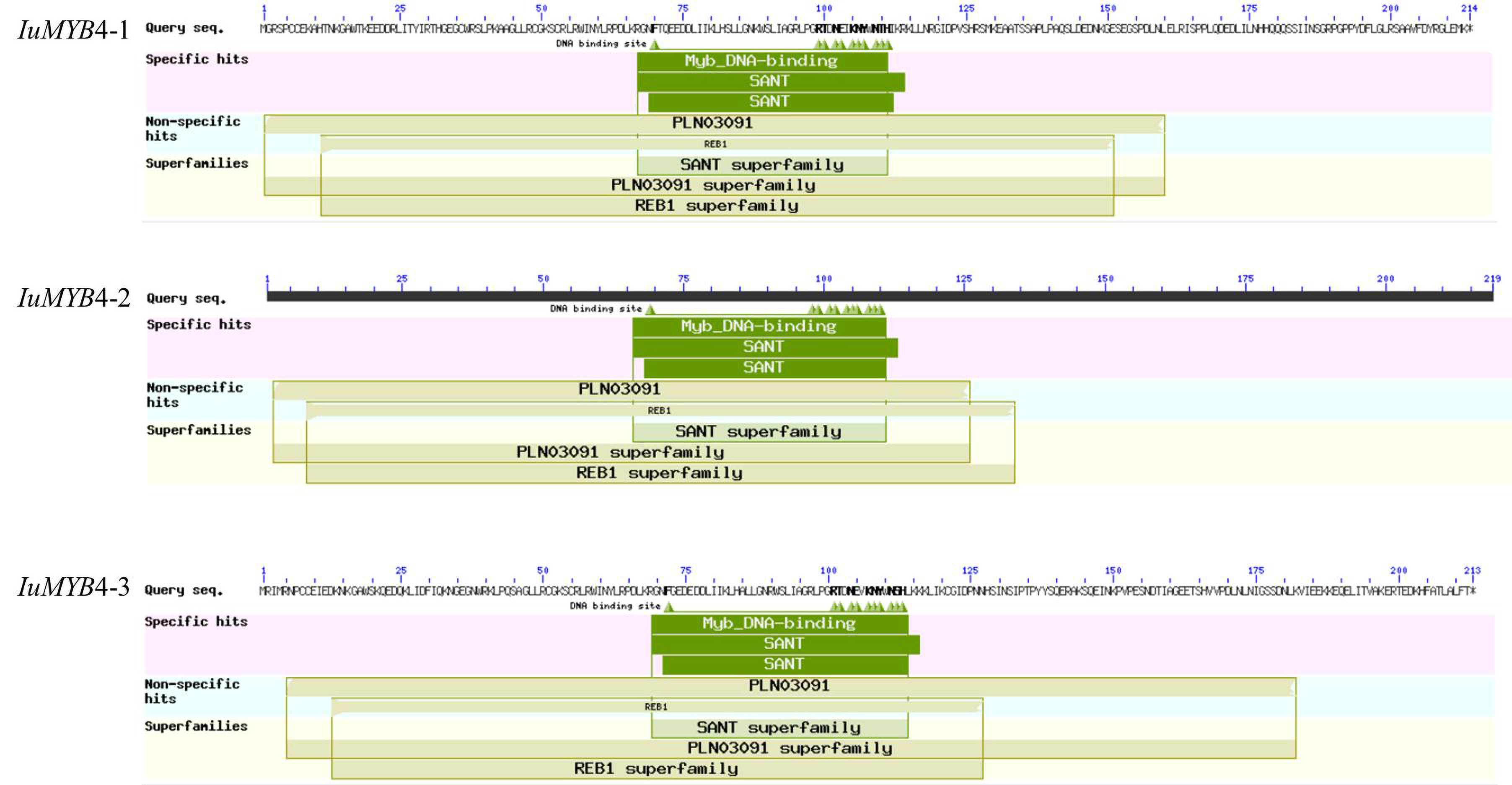


Figure S8 Conserved domains and superfamily of IuMYB4-1, IuMYB4-2 and IuMYB4-3


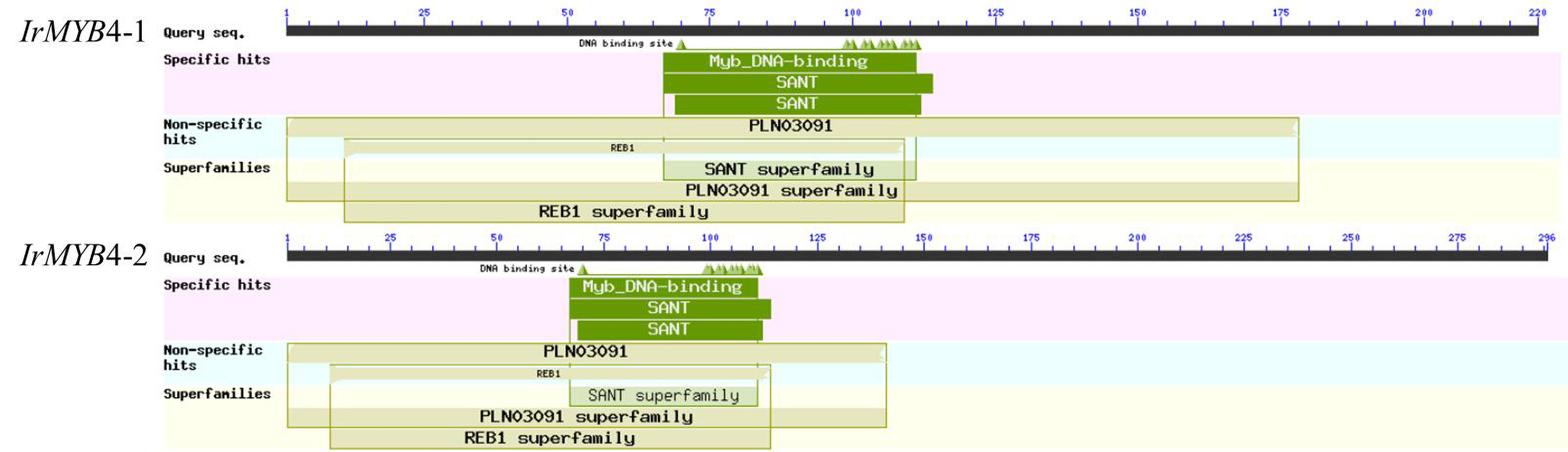


Figure S9 Prediction of Functional domain of IrMYB4-1 and IrMYB4-2.


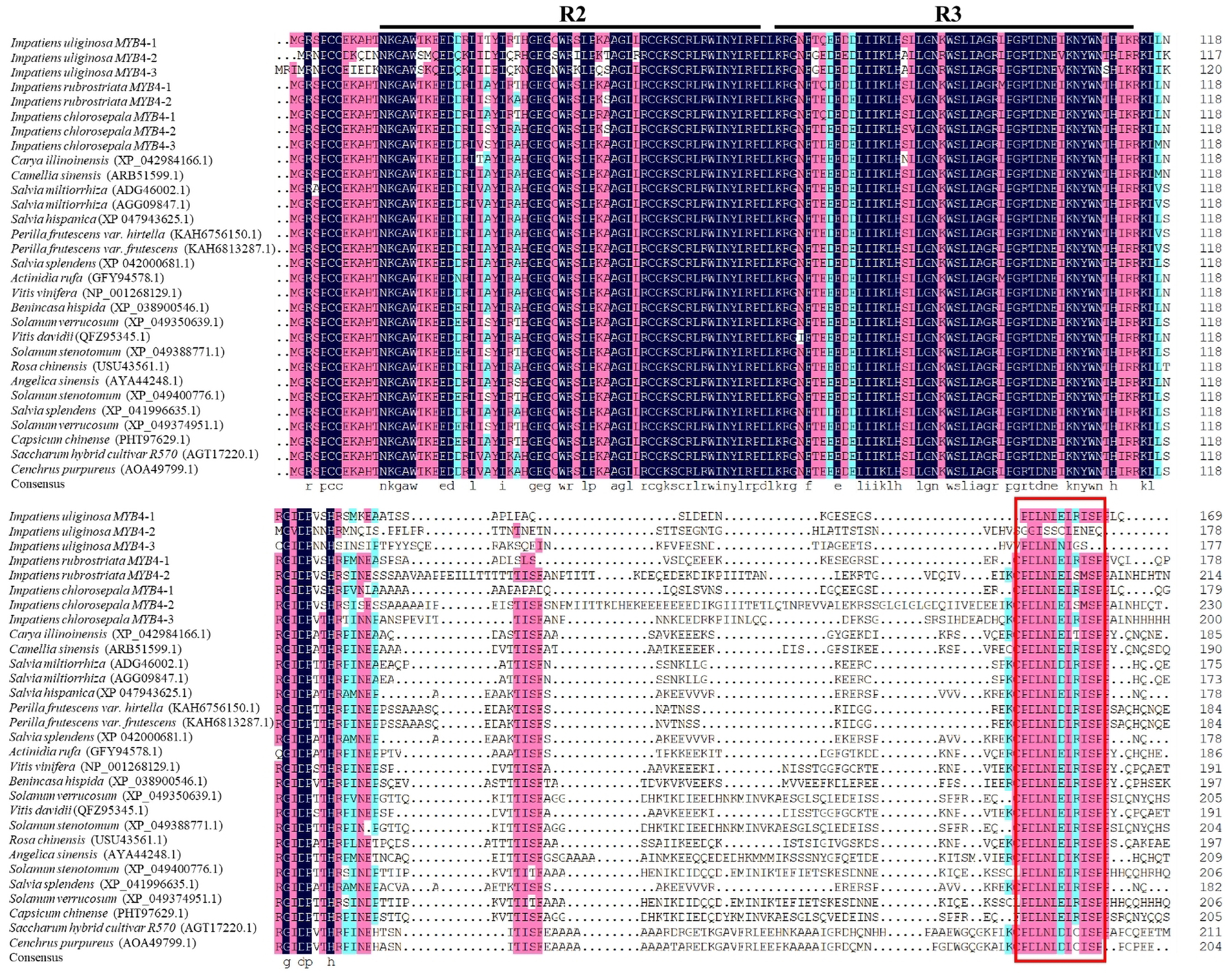


Figure S10 Homologous amino acid sequence alignment of *MYB*4 of three *Impatiens.*

The red box in the figure indicates the conservative ear motif (PDLNLELRIS); The lined sections are R2 and R3 repeats domains.

*IcMYB*4-1 cDNA:

ATGGGAAGGTCTCCCTGCTGCGAGAAAGCTCACACCAATAAAGGTGCTTGGACCAAAGAAGAAGACGACCGACTCATCGCCTACATCCGTACACACGGCGAGGGATGCTGGCGGTCCCTCCCAAGAGCCGCCGGACTCCTCCGCTGCGGCAAGAGTTGCCGACTCCGTTGGATCAATTATCTCCGACCGGATCTCAAACGCGGAAACTTCACGCAGGATGAAGACGACCTTATCATCAAATTACACAGCCTCCTCGGCAACAAATGGTCGCTGATTGCCGGAAGGTTACCGGGAAGGACTGATAATGAGATAAAAAATTATTGGAATACCCATATAAAAAGGAAGCTTTTGAACAGAGGAATCGACCCAGTTAGCCACCGCCCCGTGAACCTGGCGGCGGCGGCGGCGGCGGCGCCGGCGCCGGCTGATCAATTGCAATCACTGTCTGTTAATTCGGATGGTCAGGAAGAAGGGTCGGATGAGAGATGCCCTGACCTAAACCTGGAGCTGAGAATCAGCCCGCCGCTTCAACAGGGAGGAGACATGATGATTCTTAATCATCAGAAGAAAGAACAGAACCAATTGCCCATGATTAATAATGGCTATGACTTCTTAGGGTTGAGATCAGCCGCTGTTTTCGATTACAGAACATTGGAGATGAAATGA

*IcMYB*4-2 cDNA:

ATGGGAAGGTCTCCTTGCTGCGAGAAAGCTCACACGAACAAGGGAGCTTGGACCAAAGAAGAAGACGATCGCCTCATTTCTTACATCAGGGCCCACGGCGAAGGCTGCTGGCGATCTCTCCCCAAATCCGCCGGACTCCTCCGCTGCGGCAAGAGCTGCCGACTCCGCTGGATCAATTACCTCCGTCCCGACCTCAAGCGCGGCAATTTCACCGACGAGGAAGACGAACTCATCATCAAATTGCACAGCGTCCTAGGCAACAAATGGTCGCTTATTGCCGGAAGATTGCCTGGGAGAACAGACAATGAGATTAAAAACTACTGGAATACCCACATCAGAAGGAAGCTTCTAAACAGAGGTATAGACCCAGTGAGTCACCGCTCCATAAGTGAGTCCTCGGCTGCGGCGGCGGCGATACCGGAAATCTCCACCATATCCTTCTCCAACCCAATGATTATTACCACCAAAGATCATGAAAAGGAAGAAGAAGAAGAAGAAGAAGACATAAAAGGCATCATCATAACAGAAACTCTGCAGACGAACAGAGAAGTAGTAGCACTGGAAAAGAGATCATCAGGTTTGGGTTTGGGTTTGGGGGATCAAATAATAGTAGAAGACGAAGAAATTAAGTGCCCTGATTTGAACCTGGAGCTGAGTATGAGCCCGCCGGCGCTGAATCATGATCAAACCATAATAATAAAGGGTGCAAGCACTTTATGCTTATCATGCAGTTTGGGAATACAGAAAAGCAAGAATTGTAGTTGTAGTAGTAGTACAATCAGCAGCAGCAGCAACAATGCCTACGATTTCTTGGGGCTGAGAACCACTCATCATCATGATCATGTAGTGTTGGATTACAGAAACTTGGAGATGAAATTCAAGATCTGA

*IcMYB*4-3 cDNA:

ATGGGAAGGTCTCCTTGCTGTGAGAAAGCTCACACCAATAAGGGAGCATGGACGAAAGAAGAAGACGACCGGCTCGTTTCTTACATCCGGGCTCACGGCGAAGGCTGCTGGCGTTCCCTCCCTAAAGCCGCCGGCCTCCTCCGCTGCGGCAAGAGCTGCCGTCTCCGGTGGATCAACTACTTAAGACCCGATCTCAAACGCGGTAATTTCACCGACGAAGAAGACGAACTCATCATCAAACTCCACAGCCTTCTCGGCAACAAATGGTCGTTAATCGCGGGAAGATTACCGGGGAGAACGGATAACGAAATCAAGAATTACTGGAATACCCATATCAGAAGGAAGCTTATGAACAGGGGAATTGACCCTGTGACCCACCGGACGATCAATAATCCGGCGAATTCGCCGGAGGTCATAACCACCATATCCTTCGCGAATCCAAATAACAAAGATGAAGACAGAAAACCCATAATTAATCTACAACAGGATCCAAAATCAGGATCACGATCAATTCATGATGAGGCTGATCATCAAAAGTGCCCTGATTTGAACCTCGAGCTTAGAATCAGCCCGCCGGCACTGAATCATCATCATCATCACCGGAAAATCGACGGTGGCACCGGCAGCTTATGTTTTTCATGCAGTTTGGGGATAGAGAAAGGCAAGGATTGTAGTTGTGAATCAAGAAATAGGATAAGCTGTATTAATTCTGCAACTGCTTATGATTTCTTGGGTTTGAGAACTTACAGAAGCTTGGAGATGAAGTTCTAG

*IcMYB*4-1 gDNA (the underlined part is intron sequence):

ATGGGAAGGTCTCCCTGCTGCGAGAAAGCTCACACCAATAAAGGTGCTTGGACCAAAGAAGAAGACGACCGACTCATCGCCTACATCCGTACACACGGCGAGGGATGCTGGCGGTCCCTCCCAAGAGCCGCCGGACTCCTCCGCTGCGGCAAGAGTTGCCGACTCCGTTGGATCAATTATCTCCGACCGGATCTCAAACGCGGAAACTTCACGCAGGATGAAGACGACCTTATCATCAAATTACACAGCCTCCTCGGCAACAAGTATGATAATTTATTAGTTTTCTTAATAAAAATGTAATCTTTCTTTTTCTTGATCTTGATCTTGATCTTGTTCTTGTTCATAGATGGTCGCTGATTGCCGGAAGGTTACCGGGAAGGACTGATAATGAGATAAAAAATTATTGGAATACCCATATAAAAAGGAAGCTTTTGAACAGAGGAATCGACCCAGTTAGCCACCGCCCCGTGAACCTGGCGGCGGCGGCGGCGGCGGCGCCGGCGCCGGCTGATCAATTGCAATCACTGTCTGTTAATTCGGATGGTCAGGAAGAAGGGTCGGATGAGAGATGCCCTGACCTAAACCTGGAGCTGAGAATCAGCCCGCCGCTTCAACAGGGAGGAGACATGATGATTCTTAATCATCAGAAGAAAGAACAGAACCAATTGCCCATGATTAATAATGGCTATGACTTCTTAGGGTTGAGATCAGCCGCTGTTTTCGATTACAGAACATTGGAGATGAAATGA

*IcMYB*4-2 gDNA (the underlined part is intron sequence):

ATGGGAAGGTCTCCTTGCTGCGAGAAAGCTCACACGAACAAGGGAGCTTGGACCAAAGAAGAAGACGATCGCCTCATTTCTTACATCAGGGCCCACGGCGAAGGCTGCTGGCGATCTCTCCCCAAATCCGCCGGACTCCTCCGCTGCGGCAAGAGCTGCCGACTCCGCTGGATCAATTACCTCCGTCCCGACCTCAAGCGCGGCAATTTCACCGACGAGGAAGACGAACTCATCATCAAATTGCACAGCGTCCTAGGCAACAAGTCAGTACTACCACCACCAACTCATCATCTAATTCTTCTTCTTCATAAATATATACCCCTAATTTTTCTAACGCTAGCTTTCTCTCTCTTCATTATTACATATATAGATGGTCGCTTATTGCCGGAAGATTGCCTGGGAGAACAGACAATGAGATTAAAAACTACTGGAATACCCACATCAGAAGGAAGCTTCTAAACAGAGGTATAGACCCAGTGAGTCACCGCTCCATAAGTGAGTCCTCGGCTGCGGCGGCGGCGATACCGGAAATCTCCACCATATCCTTCTCCAACCCAATGATTATTACCACCAAAGATCATGAAAAGGAAGAAGAAGAAGAAGAAGAAGACATAAAAGGCATCATCATAACAGAAACTCTGCAGACGAACAGAGAAGTAGTAGCACTGGAAAAGAGATCATCAGGTTTGGGTTTGGGTTTGGGGGATCAAATAATAGTAGAAGACGAAGAAATTAAGTGCCCTGATTTGAACCTGGAGCTGAGTATGAGCCCGCCGGCGCTGAATCATGATCAAACCATAATAATAAAGGGTGCAAGCACTTTATGCTTATCATGCAGTTTGGGAATACAGAAAAGCAAGAATTGTAGTTGTAGTAGTAGTACAATCAGGCAGCAGCAGCAACAATGCCTACGATTTCTTGGGGCTGAGAACCACTCATCATCATGATCATGTAGTGTTGGATTACAGAAACTTGGAGATGAAATTCAAGATCTGA

*IcMYB*4-3 gDNA (the underlined part is intron sequence):：

ATGGGAAGGTCTCCTTGCTGTGAGAAAGCTCACACCAATAAGGGAGCATGGACGAAAGAAGAAGACGACCGGCTCGTTTCTTACATCCGGGCTCACGGCGAAGGCTGCTGGCGTTCCCTCCCTAAAGCCGCCGGCCTCCTCCGCTGCGGCAAGAGCTGCCGTCTCCGGTGGATCAACTACTTAAGACCCGATCTCAAACGCGGTAATTTCACCGACGAAGAAGACGAACTCATCATCAAACTCCACAGCCTTCTCGGCAACAAGTAATTCATTAACTCCTCTGTTTTTTTGTGTGTTCGATCTGTTTCTTGTTCTTGTTCTTGTTCTTGTTTCAACCCATCAATTCCAAAACCATAAAGGTTTAATCTTTTCTATTCTAGATGGTCGTTAATCGCGGGAAGATTACCGGGGAGAACGGATAACGAAATCAAGAATTACTGGAATACCCATATCAGAAGGAAGCTTATGAACAGGGGAATTGACCCTGTGACCCACCGGACGATCAATAATCCGGCGAATTCGCCGGAGGTCATAACCACCATATCCTTCGCGAATCCAAATAACAAAGATGAAGACAGAAAACCCATAATTAATCTACAACAGGATCCAAAATCAGGATCACGATCAATTCATGATGAGGCTGATCATCAAAAGTGCCCTGATTTGAACCTCGAGCTTAGAATCAGCCCGCCGGCACTGAATCATCATCATCATCACCGGAAAATCGACGGTGGCACCGGCAGCTTATGTTTTTCATGCAGTTTGGGGATAGAGAAAGGCAAGGATTGTAGTTGTGAATCAAGAAATAGGATAAGCTGTATTAATTCTGCAACTGCTTATGATTTCTTGGGTTTGAGAACTTACAGAAGCTTGGAGATGAAGTTCTAG

*IuMYB*4-1 cDNA:

ATGGGAAGGTCTCCCTGCTGCGAGAAAGCTCACACCAATAAAGGTGCTTGGACCAAAGAAGAAGACGATCGCCTCATCACCTACATCCGTACGCACGGCGAGGGATGCTGGCGTTCCCTCCCAAAAGCCGCCGGCCTCCTCCGCTGCGGCAAGAGTTGTCGCCTCCGTTGGATCAATTATCTCCGACCCGATCTCAAACGCGGAAATTTCACTCAAGAGGAAGACGATCTCATCATCAAATTGCACAGCCTCCTCGGCAACAAATGGTCGCTGATTGCCGGAAGGTTGCCCGGTAGAACAGACAACGAGATAAAGAATTATTGGAATACCCATATAAAAAGGAAGCTTCTGAACAGAGGAATAGACCCGGTTAGCCACCGGTCCATGAAAGAGGCGGCGACATCATCGGCGCCGCTTCCGGCTCAATCCCTTGATGAAGATAACAAAGGAGAATCGGAAGGAAGCCCAGACCTTAACCTGGAGCTGAGAATCAGCCCGCCGCTTCAAGACGAAGACCTAATTCTTAATCATCATCAGCAGCAGTCGTCAATAATTAATAGTGGGCGGCCGGGTCCTCCTTATGACTTTTTAGGGTTAAGATCAGCCGCTGTTTTCGATTACAGAGGCTTGGAGATGAAATGA

*IuMYB*4-2 cDNA:

ATGAGGAATCCTTGTTGCGATAAACAAGACAACAACAAAGGAGCTTGGTCCATGCAAGAAGATCAAAAGCTCATCGACTACATTCAAAGACACGGCGAAGGTTCTTGGCGCATATTACCTAAGACGGCAGGACTACGTCGTTGTGGTAAGAGTTGTAGACTTAGATGGATAAATTATCTAAGGCCCGATCTCAAGAGAGGAAATTTTGGTGAAGATGAAGAAGACCTCATCATTAAGTTACACGCACTCTTGGGAAACAGGTGGTCTTTAATTGCTGGACGACTGCCTGGAAGAACCGACAATGAAGTGAAAAACTATTGGAACACACATTTGAGAAGGAAACTTATAAAGATGGGTGTCGATCCAAACAATCACCGAATGAACCAAATAAGTCCATTTCTCCCTCGAACCACAAATACCAATGAAACTAATTCGACGACGTCCGAAGGCAACACGGGCCATCTAGCCACCACAAGTACAAGTAATGTTGATCATGTCTCCGGTGGGATAAGTTCATGTCTTGAGAATGAACAACTAAGTGTAGGTTGTCGATTGTTGCTCCCTGATCTAAACCTTGATGTTGTTGATATTGAAGAAAGAAAAACAGTAAAGGTTGTCACAACATTAATGCCTCCTACCCTTCCACTTTTTAGTTAA

*IuMYB*4-3 cDNA:

ATGAGAATCATGAGAAACCCTTGCTGCGAAATCGAAGATAAGAACAAAGGAGCTTGGTCCAAACAAGAAGATCAAAAGCTTATTGATTTCATCCAAAAGAATGGTGAAGGGAATTGGCGAAAACTCCCCCAATCTGCAGGCTTGCTTCGATGCGGTAAGAGTTGTCGGTTGAGATGGATAAACTATTTGAGGCCTGACCTTAAACGGGGTAATTTTGGAGAAGATGAAGATGACCTAATTATCAAACTCCATGCACTATTGGGAAACAGGTGGTCATTAATAGCTGGAAGACTACCCGGAAGAACAGATAACGAGGTTAAAAACTATTGGAATTCACATCTTAAGAAGAAGCTAATCAAATGTGGCATTGATCCGAATAACCATTCAATTAACAGTATCCCAACTCCATATTATTCTCAAGAACGCGCTAAATCACAAGAGATTAATAAGCCCGTTCCTGAATCAAACGACACAATAGCAGGAGAAGAAACAAGTCATGTCGTACCTGATTTGAATCTTAACATAGGATCATCCGATAATTTGAAGGTTATTGAAGAGAAGAAAGAACAAGAGTTAATTACCGTCGCAAAAGAAAGGACCGAGGACAAGCATTTTGCGACACTCGCCCTCTTCACGTAA

*IuMYB*4-1 gDNA (the underlined part is intron sequence):

ATGGGAAGGTCTCCCTGCTGCGAGAAAGCTCACACCAATAAAGGTGCTTGGACCAAAGAAGAAGACGATCGCCTCATCACCTACATCCGTACGCACGGCGAGGGATGCTGGCGTTCCCTCCCAAAAGCCGCCGGCCTCCTCCGCTGCGGCAAGAGTTGTCGCCTCCGTTGGATCAATTATCTCCGACCCGATCTCAAACGCGGAAATTTCACTCAAGAGGAAGACGATCTCATCATCAAATTGCACAGCCTCCTCGGCAACAAGTAAAATATTAATCTTTACGTCTTTTTTCCCTCTTTCCATTAAAAATCATAATCATAACTAAATTAGTTTATTTTGTTTTCTTGTTCGTAGATGGTCGCTGATTGCCGGAAGGTTGCCCGGTAGAACAGACAACGAGATAAAGAATTATTGGAATACCCATATAAAAAGGAAGCTTCTGAACAGAGGAATAGACCCGGTTAGCCACCGGTCCATGAAAGAGGCGGCGACATCATCGGCGCCGCTTCCGGCTCAATCCCTTGATGAAGATAACAAAGGAGAATCGGAAGGAAGCCCAGACCTTAACCTGGAGCTGAGAATCAGCCCGCCGCTTCAAGACGAAGACCTAATTCTTAATCATCATCAGCAGCAGTCGTCAATAATTAATAGTGGGCGGCCGGGTCCTCCTTATGACTTTTTAGGGTTAAGATCAGCCGCTGTTTTCGATTACAGAGGCTTGGAGATGAAATGA

*IuMYB*4-2 gDNA (the underlined part is intron sequence):

ATGAGGAATCCTTGTTGCGATAAACAAGACAACAACAAAGGAGCTTGGTCCATGCAAGAAGATCAAAAGCTCATCGACTACATTCAAAGACACGGCGAAGGTTCTTGGCGCATATTACCTAAGACGGCAGGACTACGTCGTTGTGGTAAGAGTTGTAGACTTAGATGGATAAATTATCTAAGGCCCGATCTCAAGAGAGGAAATTTTGGTGAAGATGAAGAAGACCTCATCATTAAGTTACACGCACTCTTGGGAAACAGTACTTATTACGGACCATCATTTCCTTTTATTTTTTAACACCTTTATTGTCAATAATAATGTGGAAAAAGATTTAAACAAAGCTTAAAATAACTTATGATCTAGAAAACACATGGACTTCTTGCTTGCTTAAAAATTCATGGGGTTAATGAACTTATAAGTGGTCTTTAATTGCTGGACGACTGCCTGGAAGAACCGACAATGAAGTGAAAAACTATTGGAACACACATTTGAGAAGGAAACTTATAAAGATGGGTGTCGATCCAAACAATCACCGAATGAACCAAATAAGTCCATTTCTCCCTCGAACCACAAATACCAATGAAACTAATTCGACGACGTCCGAAGGCAACACGGGCCATCTAGCCACCACAAGTACAAGTAATGTTGATCATGTCTCCGGTGGGATAAGTTCATGTCTTGAGAATGAACAACTAAGTGTAGGTTGTCGATTGTTGCTCCCTGATCTAAACCTTGATGTTGTTGATATTGAAGAAAGAAAAACAGTAAAGGTTGTCACAACATTAATGCCTCCTACCCTTCCACTTTTTAGTTAA

*IuMYB*4-3 gDNA (the underlined parts are intron sequences):

ATGAGAATCATGAGAAACCCTTGCTGCGAAATCGAAGATAAGAACAAAGGAGCTTGGTCCAAACAAGAAGATCAAAAGCTTATTGATTTCATCCAAAAGAATGGTGAAGGGAATTGGCGAAAACTCCCCCAATCTGCAGGTTCAAATCATTTCTTTTCGTATATTTGTTCATCGGATGAAAACAATCTTATTTATATCTCGTGAATCAGGCTTGCTTCGATGCGGTAAGAGTTGTCGGTTGAGATGGATAAACTATTTGAGGCCTGACCTTAAACGGGGTAATTTTGGAGAAGATGAAGATGACCTAATTATCAAACTCCATGCACTATTGGGAAACAGGTGCCTACTAATTTCAATTATCATATTCCATATATACTAGACTTCTTCTCTTAAAATTTATGTAAAATTTTCTTTTAATATATTTTAAATAGATTGTTCATAATTATGAATTAGGTGGTCATTAATAGCTGGAAGACTACCCGGAAGAACAGATAACGAGGTTAAAAACTATTGGAATTCACATCTTAAGAAGAAGCTAATCAAATGTGGCATTGATCCGAATAACCATTCAATTAACAGTATCCCAACTCCATATTATTCTCAAGAACGCGCTAAATCACAAGAGATTAATAAGCCCGTTCCTGAATCAAACGACACAATAGCAGGAGAAGAAACAAGTCATGTCGTACCTGATTTGAATCTTAACATAGGATCATCCGATAATTTGAAGGTTATTGAAGAGAAGAAAGAACAAGAGTTAATTACCGTCGCAAAAGAAAGGACCGAGGACAAGCATTTTGCGACACTCGCCCTCTTCACGTAA

*IrMYB*4-1 cDNA:

ATGGGAAGGTCTCCCTGCTGCGAGAAAGCTCACACCAACAAAGGAGCTTGGACCAAAGAAGAAGACGATCGACTCATCGCTTACATCCGTACGCACGGCGAGGGATGCTGGCGCTCCCTCCCGAAAGCTGCCGGACTCCTCCGCTGCGGCAAGAGCTGCCGCCTCCGTTGGATCAATTATCTCCGGCCAGATCTCAAGCGCGGAAATTTCACGCAAGATGAAGATGACCTTATCATCAAATTACACAGCCTCCTCGGCAACAAATGGTCGCTGATTGCGGGAAGGATGCCGGGTAGAACGGATAATGAGATAAAAAATTATTGGAATACCCATATAAAAAGGAAGCTTTTGAACAGAGGAATAGACCCGGTTAGCCACCGCCCCATGAACGAGGCGTCGCCGTCGGCGGCGGATCTATCCCTCTCAGTTTCCGATCAGGAAGAAGAGAAGAAAGAATCGGAAGGAAGGTCAGATGAGAGATGCCCTGACCTAAACCTGGAGCTGAGAATCAGCCCGCCGGTTCAACTTCAACCGGCAGATGACCTAATTCTTAATAGTGGGCTGCCGGTTCCGATTCCGGTGCCGGGTCCGCTTGCTTATGACTTCTTAGGGCTGAGATCAGCCGCTGTTTTCGATTATAGAAGTTTGGAGATGAAATGA

*IrMYB*4-2 cDNA:

ATGGGAAGGTCTCCTTGCTGTGAGAAAGCTCACACCAACAAGGGAGCTTGGACCAAAGAAGAAGACGATCGCCTCATTTCTTACATCAAGGCCCACGGCGAAGGCTGCTGGCGATCCCTCCCCAAATCCGCTGGACTCCTCCGCTGCGGAAAGAGCTGCCGACTCCGCTGGATCAATTACCTCCGTCCCGACCTCAAGCGCGGCAATTTCACCGAGGATGAAGACGAACTCATCATCAAATTGCACAGCGTCCTAGGCAACAAATGGTCGCTTATTGCCGGGAGACTGCCGGGGAGAACAGACAACGAGATTAAAAATTACTGGAATACCCACATCAGAAGGAAGCTTCTCAACAGAGGTATAGACCCAGTAAGTCACCGCTCCATAAACGAGTCATCGTCCGCGGCTGTGGCGGCACCACCGGAAATCTTGTTGACCACCACCACTACCACCATATCTTTCGCCAACCCAACGATTACAACCAAAGATGAACAAGAAGATGAAAAAGACATAAAACCCATCATCATAACAGCAAATCTGGAAAAGAGAACAGGGGTGGATCAAATAGTAGAGATTAAGTGCCCTGATTTGAACCTGGAGCTTAGTATGAGCCCGCCGGCTCTCAATCACGATCATACAAATACAATTGTAAAGGGTACAAGCCCCCCTTTATGCTTTTCATGCAGTTTGGGGATACAGAAAAGCAAGGATTGTAGTTGTAGCAGCAGCAGCATAAGTAGTAATAGTAGTAACAGTAGTAGTACAATCAGCAGCAGCAATAACAGCAATGCTTACGATTTCTTGGGGTTGAGAACTACTCATCACCATGATCATGATCATCATGTAATGTTGGATTACAGAAGCTTGGAGATGAAGTTCAAGATCTGA

*IrMYB*4-1 gDNA (the underlined part is intron sequence):

ATGGGAAGGTCTCCCTGCTGCGAGAAAGCTCACACCAACAAAGGAGCTTGGACCAAAGAAGAAGACGATCGACTCATCGCTTACATCCGTACGCACGGCGAGGGATGCTGGCGCTCCCTCCCGAAAGCTGCCGGACTCCTCCGCTGCGGCAAGAGCTGCCGCCTCCGTTGGATCAATTATCTCCGGCCAGATCTCAAGCGCGGAAATTTCACGCAAGATGAAGATGACCTTATCATCAAATTACACAGCCTCCTCGGCAACAAGTAAGCCCCTTGTCCCGTTTGAGACTTTTAAAAGTTAATTATTTATTTATTAATTAAAAAAAAATGTTTTTGTGGTTCATAAATGGTCGCTGATTGCGGGAAGGATGCCGGGTAGAACGGATAATGAGATAAAAAATTATTGGAATACCCATATAAAAAGGAAGCTTTTGAACAGAGGAATAGACCCGGTTAGCCACCGCCCCATGAACGAGGCGTCGCCGTCGGCGGCGGATCTATCCCTCTCAGTTTCCGATCAGGAAGAAGAGAAGAAAGAATCGGAAGGAAGGTCAGATGAGAGATGCCCTGACCTAAACCTGGAGCTGAGAATCAGCCCGCCGGTTCAACTTCAACCGGCAGATGACCTAATTCTTAATAGTGGGCTGCCGGTTCCGATTCCGGTGCCGGGTCCGCTTGCTTATGACTTCTTAGGGCTGAGATCAGCCGCTGTTTTCGATTATAGAAGTTTGGAGATGAAATGA

*IrMYB*4-2 gDNA (the underlined part is intron sequence):

ATGGGAAGGTCTCCTTGCTGTGAGAAAGCTCACACCAACAAGGGAGCTTGGACCAAAGAAGAAGACGATCGCCTCATTTCTTACATCAAGGCCCACGGCGAAGGCTGCTGGCGATCCCTCCCCAAATCCGCTGGACTCCTCCGCTGCGGAAAGAGCTGCCGACTCCGCTGGATCAATTACCTCCGTCCCGACCTCAAGCGCGGCAATTTCACCGAGGATGAAGACGAACTCATCATCAAATTGCACAGCGTCCTAGGCAACAAATCAGTCACTCCCTCCCCTGCTTCCTTCTTCTATCACTCACTCACGTTTCCTTTCCCTGGTTTTCCTTCATACCGAAGGTCGCTTATTGCCGGGAGACTGCCGGGGAGAACAGACAACGAGATTAAAAATTACTGGAATACCCACATCAGAAGGAAGCTTCTCAACAGAGGTATAGACCCAGTAAGTCACCGCTCCATAAACGAGTCATCGTCCGCGGCTGTGGCGGCACCACCGGAAATCTTGTTGACCACCACCACTACCACCATATCTTTCGCCAACCCAACGATTACAACCAAAGATGAACAAGAAGATGAAAAAGACATAAAACCCATCATCATAACAGCAAATCTGGAAAAGAGAACAGGGGTGGATCAAATAGTAGAGATTAAGTGCCCTGATTTGAACCTGGAGCTTAGTATGAGCCCGCCGGCTCTCAATCACGATCATACAAATACAATTGTAAAGGGTACAAGCCCCCCTTTATGCTTTTCATGCAGTTTGGGGATACAGAAAAGCAAGGATTGTAGTTGTAGCAGCAGCAGCATAAGTAGTAATAGTAGTAACAGTAGTAGTACAATCAGCAGCAGCAATAACAGCAATGCTTACGATTTCTTGGGGTTGAGAACTACTCATCACCATGATCATGATCATCATGTAATGTTGGATTACAGAAGCTTGGAGATGAAGTTCAAGATCTGA
